# Successive Cambia: A Developmental Oddity or an Adaptive Structure?

**DOI:** 10.1371/journal.pone.0016558

**Published:** 2011-01-31

**Authors:** Elisabeth M. R. Robert, Nele Schmitz, Ilse Boeren, Tess Driessens, Kristof Herremans, Johan De Mey, Elke Van de Casteele, Hans Beeckman, Nico Koedam

**Affiliations:** 1 Laboratory for Plant Biology and Nature Management (APNA), Vrije Universiteit Brussel (VUB), Brussels, Belgium; 2 Laboratory for Wood Biology and Xylarium, Royal Museum for Central Africa (RMCA), Tervuren, Belgium; 3 Institute of Botany (210), University of Stuttgart-Hohenheim, Stuttgart, Germany; 4 Radiology, Universitair Ziekenhuis Brussel, Brussels, Belgium; 5 SkyScan NV, Kontich, Belgium; 6 Vision Lab, Universiteit Antwerpen (UA), Wilrijk, Belgium; Purdue University, United States of America

## Abstract

**Background:**

Secondary growth by successive cambia is a rare phenomenon in woody plant species. Only few plant species, within different phylogenetic clades, have secondary growth by more than one vascular cambium. Often, these successive cambia are organised concentrically. In the mangrove genus *Avicennia* however, the successive cambia seem to have a more complex organisation. This study aimed (i) at understanding the development of successive cambia by giving a three-dimensional description of the hydraulic architecture of *Avicennia* and (ii) at unveiling the possible adaptive nature of growth by successive cambia through a study of the ecological distribution of plant species with concentric internal phloem.

**Results:**

*Avicennia* had a complex network of non-cylindrical wood patches, the complexity of which increased with more stressful ecological conditions. As internal phloem has been suggested to play a role in water storage and embolism repair, the spatial organisation of *Avicennia* wood could provide advantages in the ecologically stressful conditions species of this mangrove genus are growing in. Furthermore, we could observe that 84.9% of the woody shrub and tree species with concentric internal phloem occurred in either dry or saline environments strengthening the hypothesis that successive cambia provide the necessary advantages for survival in harsh environmental conditions.

**Conclusions:**

Successive cambia are an ecologically important characteristic, which seems strongly related with water-limited environments.

## Introduction

**Figure 1:**
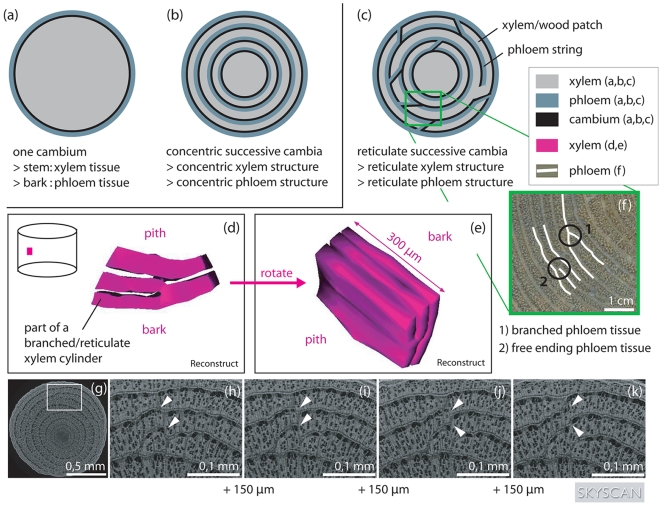
Spatial structure of the xylem and phloem tissue in *Avicennia* in comparison to other trees. Schematic view of a stem disc from (a) a tree with only one cambium, (b) a tree with successive cambia organised in concentric cylinders, giving rise to a stem disc with concentric circles of xylem tissue, phloem tissue and cambium circles and (c) *Avicennia*, having a reticulate organisation of its cambia and transport structure. The spatial structure of *Avicennia* is depicted by smoothed surface images after three-dimensional reconstruction in Reconstruct [42] (d,e). Only a small part of the xylem tissue has been visualised. It can be seen that *Avicennia* had a complex network of xylem patches that fused at certain heights of the tree and joined different patches at other parts of the stem. The connections between xylem patches changed rapidly with vertical distance. This is shown by the white arrowheads in the four serial micro-CT-scan images of an outside zone of an *Avicennia marina* stem disc, produced by a SkyScan 1172 scanner (g-k). Each section is 150 µm apart from the previous. On the micro-CT-images (g-k) the wide light grey bands are xylem tissue while the dark small strings are phloem tissue. The complexity of the internal structure can be expressed by the sum of the locations of free ending and branched phloem tissue (f) per stem disc surface area.

Expansion in girth in most vascular plant species is the result of the meristematic activity of one cylindrical vascular cambium, producing xylem towards the inner and phloem towards the outer part of the plant stem (Fig. 1a) [1]. However, some plant species (out of 34 families according to the taxonomy used [1]) have not one but several successive cambia causing the secondary growth and the resulting wood anatomy to be different from that of plants with only one vascular cambium (Fig. 1b). In the stem of plants with successive cambia a sequence of vascular cambia can be found, each responsible for the production of secondary xylem inwards and secondary phloem outwards (Fig. 1b) [2]. The cambia can literally develop successively but several cambia can also develop simultaneously [3]. This results in plants from which stem discs show a succession of dark coloured xylem tissue bands and pale coloured phloem tissue bands.

Special forms of secondary wood formation are typical for lianas and vines [e.g. 1], [4], [5], [6], [7], though the phenomenon of successive cambia is also found in a select group of herb, shrub and tree species [e.g. 1], [8], [9], [10], [11], [12] coming from different phylogenetic clades [1], [2], [12]. The genus *Avicennia* is the only mangrove tree genus showing the developmental oddity of secondary growth by successive cambia. Xylem and phloem tissue in *Avicennia* are found to be non-concentric (Fig. 1c) [e.g. 3], [13], [14], [15], [16], [17], [18] and form an intricate three-dimensional network instead of separate concentric cylinders [3], [13], [15]. Detailed and four dimensional - the three spatial dimensions and time - observations of these xylem and phloem networks are lacking, though highly necessary to understand *Avicennia*'s growth pattern and the secondary growth of trees with successive cambia in general.

From a study in two dimensions, the nature of secondary growth in *Avicennia marina* has been suggested to be patch-like, with active growth displacing around the stem circumference with time [3]. In *Avicennia*, cambium differentiation was found to be internally controlled [13], [14], [19], with indications for an interaction with the micro-environmental conditions such as soil water salinity [3]. What triggers the development of new vascular cambia however remains unclear. In the harsh and changing environmental conditions of the mangrove habitat, the secondary growth of *Avicennia marina* and the resulting pattern of vascular tissue have been proposed to offer a functional advantage for the water transport. While the non-lignified and more thin-walled phloem tissue in between the more rigid xylem tissue increases the mechanical flexibility of lianas [1], [2], [20], it can also provide water and photosynthate supply [3], [13], [21], [22], [23], [24] for plants growing under water stress. The living phloem tissue in at least the outer part of the stem could play an important role in embolism repair [21], [22], [23], [24], [25], [26]. In addition, beneficial growth conditions can be exploited by simultaneous activity of more than one cambium or by alternating cambial activity around the stem disc. In this way, wood segments with different wood anatomical characteristics – and thus different physiological possibilities - can be created around the stem disc.

In this study we aimed at clarifying the three-dimensional structure of the xylem and phloem tissue of the mangrove genus *Avicennia* through (micro-)CT-scanning. X-ray (micro-)CT scanning allows a non-destructive visualization of the internal structures at high resolution. In order to differentiate between connected structures a difference in X-ray density is required. Since xylem is denser than phloem tissue, this technique is appropriate to study the 3D structure. We further investigated the effect of the environment on the spatial organisation of this structure to unveil aspects of the remarkable adaptation of *Avicennia* to the mangrove ecosystem. In order to widen the scope of our conclusions, we tested the relation between habitats that are susceptible to water stress and the presence of woody species with concentric internal phloem. As successive cambia and the resulting internal phloem rings are thought to bring physiological advantage in harsh environmental conditions, we hypothesized that the major part of the woody species with internal concentric phloem are growing in habitats that are physiologically harsh either by drought or salt stress.

## Results

### The Three-Dimensional Structure of *Avicennia'*s Transport System

A reticulate xylem structure, consisting of xylem patches joined in horizontal as well as in vertical direction (Fig. 1c–k), was found consistently in all species and tree samples studied. Within this reticulate system, the connections between xylem zones (patches) changed rapidly with height (Fig. 1h–k) creating a complex network of xylem patches that fused at certain heights of the tree but separated to join different patches in other parts of the tree stem (Fig. 1d–e). In small zones of wood, it could be observed that vessels, although in the same organisation with respect to each other, were more or less scattered depending on height. The remaining space was filled with fibre cells. Moreover, xylem volumes with their corresponding phloem could be found to belong to one growth patch at a certain tree height, while being part of another one at another height (Fig. 1h–k). Therefore phloem and xylem parts that were not connected and part of different growth patches at a certain place in the tree stem lined up lower or higher in the tree.

Phloem was observed to have the same reticulate structure as the xylem network: in most parts of the stem discs studied phloem did not form concentric rings but an assembly of free ending strings and branched circles (Fig. 1f–k). Considering this in three dimensions, we could recognize free ending sheets and branched cylinders, the three-dimensional equivalents of free ending tissue and branched circles respectively (Fig. 1d,e). At certain heights of the tree stems, phloem was found to exist in very small portions that were not connected with the surrounding phloem network at that same height. In these parts of the tree a continuous circular band of phloem around the stem circumference was lacking. In a part of the stem of the *A. germinans* individual from Benin (Tw50689, Table 1) small isolated patches of xylem completely surrounded by phloem tissue were observed on the transverse CT-images. They were at least 4.56 cm in height.

**Table 1:** Overview of the *Avicennia* samples used for the CT-analysis and micro-CT- analysis.

species	collection number	country	location	biogeographical region	analysis
*A. marina*	Tw58927-9	Kenya	Gazi Bay – site 1	eastern	CT
	Tw58916, 18, 19	Kenya	Gazi Bay – site 2	eastern	CT
	Tw58937, 38, 41	Kenya	Gazi Bay – site 3	eastern	CT
	Tw60819	Kenya	Gazi Bay – site 3	eastern	CT*
	Tw60820	Kenya	Gazi Bay – site 2	eastern	CT**
	Tw60821	Kenya	Gazi Bay	eastern	micro-CT
	Tw42907	Union of the Comoros	Ngasidja	eastern	CT
	Tw57257	Sri Lanka	Rekawa	eastern	CT
*A. officinalis*	Tw57246-8	Sri Lanka	Pambala	eastern	CT
	Tw57255	Sri Lanka	Rekawa	eastern	CT
*A. germinans*	Tw50689	Benin	Cotonou	western	CT
	Tw57679	Democratic Republic of Congo	Ile Bula	western	CT
	Tw55845	Democratic Republic of São Tomé and Príncipe	Lagoa Azul	western	CT

Samples were selected from the xylarium of the Royal Museum of Central Africa in Tervuren (Belgium) or collected in the field (Gazi Bay – Kenya).

* (root sample),

** (root sample and samples at different heights).

Vessels were found to be rather straight at distances of less than 6.5 mm. However, we observed, at zones of the stem disc with a highly reticulate transport system, that some vessels were part of a certain wood patch at a certain height of the tree but were part of wood volumes that were non-existing at this height, only a few hundred micrometers higher in the tree.

**Figure 2:**
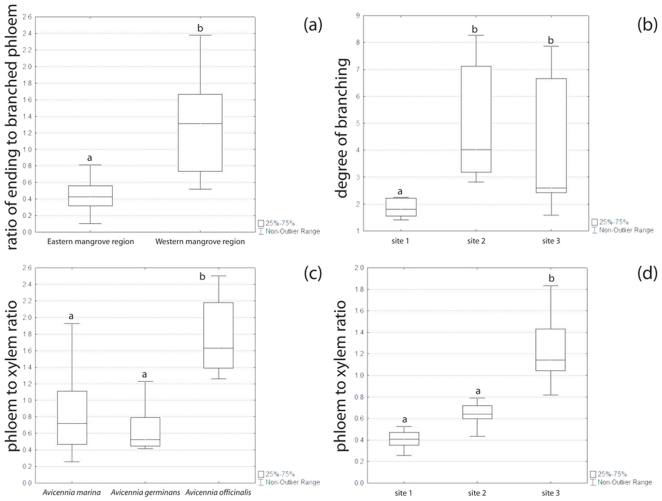
Characteristics of the spatial network of the transport system in *Avicennia*. (a) Ratio of ending to branched phloem in function of the biogeographical mangrove regions; (b) Degree of branching of the transport network of *Avicennia marina* in function of the study site (Table 2); (c–d) Ratio of phloem surface area to wood surface area in function of the mangrove species (c) and in function of the study site (Table 2) for *A. marina* (d). Lines: medians. Different letters indicate significant differences.

The ratio of ending to branched phloem (Fig. 1f) depended on species (H = 19.64, p<0.001, Kruskal-Wallis test) but not on the ecological conditions of the growing site (*A.* marina – Kenya; H = 2.04, p>0.05, Kruskal-Wallis test). The species of the eastern biogeographic mangrove region (Indo-West Pacific and East Africa) had a significantly lower ratio than *A. germinans* of the western biogeographic mangrove region (America and West Africa) (U = 32.50, p<0.001, Mann-Whitney U test, Fig. 2a). On the contrary, the level of branching of the xylem and phloem network, expressed as the number of points were the growth segments are not concentric per surface area, did not depend on species (H = 1.99, p>0.05, Kruskal-Wallis test) but on growing site (H = 16.67, p<0.001, Kruskal-Wallis test, Fig. 2b). The lowest values were found for trees growing in the study site with the lowest salinity and the highest inundation frequency (Table 2). There was a significant difference in ratio of phloem surface area to wood surface area in *A. officinalis* as compared to *A. marina* and *A. germinans* (H = 21.49, p<0.001, Kruskal-Wallis test, Fig. 2c). Within *A. marina* the growing site had a significant effect on the phloem to xylem ratio (H = 19.83, p<0.001, Kruskal-Wallis test, Fig. 2d).

**Table 2:** Stand characteristics of *Avicennia marina* study sites in Gazi Bay, Kenya [3].

Location			Soil Water Nutrients (10^−3^ µmol/cm^3^ soil)									LAI	Soil texture	Soil Water Salinity (‰)			Inundation class
	°S	°E	NO_3_^−^			NH_4_^+^			P					mean	min	max	
			mean	min	max	mean	min	max	mean	min	max						
site 1	4°24′54,0″	39°30′42,0″	0.5	0.2	0.6	3	0	5	1.4	0.9	1.8	1.42	silty clay	34.6	20.9	46.0	I
site 2	4°25′55,5″	39°30′34,5″	0.15	0.10	0.24	*	*	*	0.2	0.1	0.3	0.23	loamy sand	66.1	38.0	86.0	III
site 3	4°25′16,0″	39°30′27,0″	2	0	6	10	7	14	0.1	0	0.3	1.18	clay loam	63.3	40.0	79.9	III

Soil water for nutrient and salinity analyses was taken at about 25 cm depth. Inundation classes are defined according to Tomlinson [44].

P (soluble reactive phosphor), LAI (Leaf Area Index),

* (no data records).

**Figure 3:**
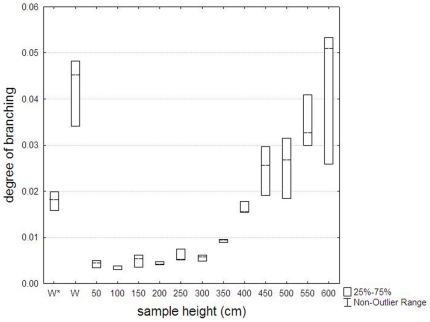
Degree of branching of the internal transport structure of *Avicennia* in function of tree height. Data are from one *Avicennia marina* tree from Gazi Bay (Kenya). Lines: medians; W: main root of the same tree; W*: main root of another *A. marina* tree from a different study site in the same mangrove forest.

Furthermore, tree height had a significant effect on the branching of the xylem and phloem tissue (H = 38.63, p<0.001, Kruskal-Wallis test): from the base to the upper crown of the tree, the degree of branching of the xylem and phloem tissue was increasing (Fig. 3). In the roots however, the degree of branching was similar to that in the crown of the tree (Fig. 3). In contrast, no trend with tree height could be found in the ratio of ending to branched phloem or in the phloem to xylem ratio.

### Distribution of concentric internal phloem species according to drought stress intensity

From the investigated woody species with concentric internal phloem, 39.6% could be found in habitats characterised with drought or salt stress while 60.4% could be found in rather wet habitats (Table 3). Then, again, the abundance of lianas was much higher in the non-stressed compared to the drought stressed category (49.5% versus 4.1% respectively) (Table 3). If lianas were excluded, 76.7% of the species was growing in dry conditions while 23.3% was growing in a wet, non-saline environment (Table 3). Ten of the fourteen shrub species growing without drought stress could also have a liana habitus. Excluding them was giving a balance of 84.9% of species in a dry and/or saline environment versus 15.1% in wet, non-saline conditions.

**Table 3:** Table showing the number of woody species with concentric internal phloem (Table S1) that occur in (periodically) dry and/or saline environments (stress) and in wet, non-saline environments (no stress) categorized by habit (lianas, shrubs and trees).

	Stress	No stress
Lianas	9 (4.1%)	110 (49.5%)
Shrubs	48 (21.6%)	14 (6.3%)
Trees	31 (14.0%)	10 (4.5%)
	88 (39.6%)	134 (60.4%)

Absolute numbers with percentage between brackets.

## Discussion

### The three-dimensional structure of *Avicennia* wood

Seen in three dimensions, the transport tissues in *Avicennia* had a network-like structure (Fig. 1c–k). This network of xylem and phloem tissue, extensively connected in horizontal and vertical direction, confirmed the existence of the structure proposed by Schmitz *et al.* [3], [13] for *A. marina* as well as with the structure proposed by Zamski [15] who studied young *A. resinifera* and *A. germinans* trees. Our study however, added that a similar three-dimensional structure systematically occurred in stems of different *Avicennia* species from different origins, and in roots, branches and stems from *A. marina* (Table 1). Therefore, the structure could be considered a general characteristic of the mangrove genus *Avicennia*. Furhtermore we added that the observed network was highly variable with tree height and position in the tree for all species studied as the connection pattern could change within a few micrometers only (Fig. 1d–e, h–k).

Similar reticulate three-dimensional structures have, as far as we are aware, been proposed (i) in small stems of *Anabasis articulata* and *Kochia indica* [11], (ii) in roots of *Beta vulgaris* [27] and (iii) in branches and stems of *Dalbergia paniculata* [28] having concentric rings of successive cambia. Furthermore, this structure has been observed in branches of *Bougainvillea spectabilis* [29], in stems of *Phytolacca dioica* [30] and in small stems of *Atriplex halimus* [8], [11] and *Haloxylon salicornicium* [11] having concentric rings of collateral vascular bundles. With the exception of Fahn *et al.* [8], [11] and Zamski 1979 [15], three-dimensional visualisations and characterisations of these structures are lacking. In general, a three-dimensional way of interpreting internal phloem is not yet developed, although the articles of Fahn and Zamski were published in 1967, 1979 and 1986 already. This lack of spatial insight is obstructing in depth discussions on the ecological and developmental implications of the observed reticulate structures.

Observation of transversal sections of other species in which successive cambia have been described as concentric rings [e.g. 27], [31], [32] showed that many more species potentially have a complex network of transport tissues. Connections between rings could already be seen in two-dimensional observations. Furthermore, in the stem of *Combretum nigricans*, the diffuse internal phloem, seen as scattered patches on transversal stem sections, was also observed to be organised as a network if watched in three dimensions [33]. This only strengthened the demand for three-dimensional observations of plant anatomy and morphology and for wood anatomical studies with three-dimensional approaches of scientific questions.

Within the observed structure, vessels were criss-crossing different xylem patches along the tree stem. Since each xylem patch is formed by another (part of the) cambium, vessel elements of the same vessel were not always derived from one cambial zone only. To assure the longitudinal alignment of vessel elements, a xylogenic signal might be given by the developing vessel to an undifferentiated cell in the cambial zone of a contiguous xylem patch via parenchyma-vessel pits [34], [35], [36].

The complex reticulate structure raised questions about its formation and functioning. What type of cambium can give rise to this kind of spatial structure? How does such a cambium originate? Is there a different system of molecular signalling in these non-continuous tissues than in a cylindrical cambium? Has this kind of organisation a functional significance? Answers to these questions touch upon our fundamental understanding of the hydraulic architecture and its ontogeny in plants and in trees in particular and may reveal unknown mechanisms of plant growth and functioning.

**Figure 4:**
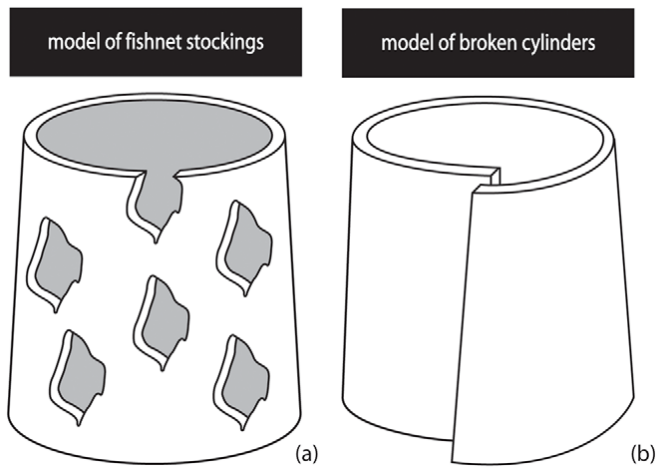
Nature of the successive cambia in *Avicennia*: two models. (a) Model of the *fishnet stockings*: multiple non-continuous cambial sheets give rise to the reticulate transport system. (b) Model of the *broken cylinders*: complete cambium shields have the ability to break at certain locations and can eventually line up in a later state of the development of the transport structure. Only one cambial layer is shown in both visualisations.

Looking at the visualized three-dimensional structure of the xylem and phloem tissue in *Avicennia*, we would expect the cambia in *Avicennia* to have the same reticulate structure as the xylem and phloem tissue, and, in that sense, be non-continuous, either in space or in time. Speculating about the nature of such cambia, we could define two possibilities: cambia that look like *fishnet stockings* or cambia that are *broken cylinders* (Fig. 4). In the first model (Fig. 4a), we imagined multiple layers of cambium sheets that are discontinuous along as well as around the tree stem and that are possibly connected, while in the second one (Fig. 4b), continuous cambium sheets have the ability to break up longitudinally and/or radially at certain locations, after formation. Both models have to be considered together with the possibility of cambia to line up radially and longitudinally once active and of different growth speeds in distinct parts of the tree as has been already proposed by Schmitz *et al.* [3].

The first as well as the second model on the three-dimensional structure and functioning of *Avicennia*'s cambia are both compatible with the proposed parenchymatic origin of new cambia in *Avicennia* [3], [13], [14], [15], [18], [30], [37]. In the *fishnet stockings* model the parenchyma cells of the assumed cambial cylinder do not all dedifferentiate to meristematic cells, giving rise to the suggested reticulate structure. This has already been proposed for *Dalbergia paniculata*, a tree species with successive cambia [28]. In the *broken cylinders* model on the other hand, modifications of the cambia, initially organised in concentric cylinders, would occur after dedifferentiation. In reality, a combination of the two proposed models is not excluded. In that case, cambia would be reticulate while formed as well as able to break at certain locations. However, only growth experiments on high spatial and temporal resolution can illuminate the exact ontology of the transport system in *Avicennia*.

Several authors report a number of simultaneously active cambia in plant species with successive cambia [3], [13], [15], [32], [38]. We should not picture different cambial zones as separate unities only because they seem to be unconnected on transverse (thin) sections. With the three-dimensional structure in mind, different zones of the cambium in different rings might be part of one and the same connected cambial zone. In that sense, the cambia, as well as the resulting transporting tissues have to be considered as co-functioning in a unit, as has been proposed for the rings of transport tissue in *B. spectabilis* by Zamski [29].

### The Ecological Advantage of Successive Cambia

Within *Avicennia* we observed a higher degree of branching in the spatial structure of the transport system in trees growing in ecological conditions that are more demanding for the water transport, *i.e.* higher salt concentrations in the soil water (mean soil water salinity and soil water salinity range) and low inundation frequency [3], [39] (Fig. 2b and Table 2). Furthermore, a higher phloem to xylem tissue ratio has been measured in the trees of the ecologically most stressful study site (Fig. 2d and Table 2). These observations corroborated the current idea for a role of phloem tissue in embolism repair [21], [22], [23], [24], [25], [26]. The more extensive branching of the phloem tissue, going with a higher absolute amount of internal phloem, as well as the higher phloem to xylem ratio might thus provide a safer water transport system, next to the enhanced water storage potential [21], [22], [23], [24].

With increasing height aboveground, the degree of branching of the transport system within an *Avicennia* tree became higher in absolute value and bigger in range. In the roots, the degree of branching was similar to that of the crown (Fig. 3). This means that in the crown and in the roots, the phloem tissue was more entangled within the xylem, leading to closer contact between the two transport tissues. In the crown an increased tension on the water column exists, and thus putting a higher demand on the water transport system requiring a higher safety level. Although the tension is lowest in the roots, securing water flow is as essential at the start of the pipeline as it is in the higher parts of the crown.

Surprisingly, the ratio of ending to branched phloem differed between species, with a lower ratio in species from the eastern biogeographical mangrove region than in *A. germinans* from the western biogeographical mangrove region (Fig. 2a). This characteristic seemed to be species-specific and therefore part of the evolutionary background of the species rather than a flexible response to environmental drivers.

Supporting the findings in *Avicennia* spp. and the functional role for the phloem in safeguarding the water transport was that 84.9% of trees and shrubs with concentric internal phloem grow in dry or saline habitats. This relationship, however, held only when lianas were excluded from the database since they were far more distributed in wet habitats (Table 3). Concerning the functional advantage concentric internal phloem - often deriving from successive cambia - might offer, a distinction has to be made between trees and shrubs on one side and lianas on the other side. In trees and shrubs, the main functional advantage of internal phloem is thought to be water and photosynthate storage as well as the role in embolism repair. For scandent lianas however, the succession of the rigid xylem tissue with the thin-walled phloem tissue provides the necessary flexibility [1], [2], [20]. Besides, the additional starch storage capability of the successive phloem layers offers an advantage for quick growth to the top of the canopy [2]. In terms of our hypothesis, it means that concentric internal phloem in lianas is not *per se* related to drought or salt stress conditions, whereas it is in trees and shrubs.

Apart from the majority of trees and shrubs with concentric internal phloem growing under drought stress conditions, there was still 15.1% related to habitats with adequate water supply. Although speculative, this can be explained by the wide range of habitats these species occur in, or by the evolutionary evolvement of these species from species in dryer habitats together with the neutral effect of growth via successive cambia on the survival of trees and shrubs growing under wet conditions. Another explanation is the higher flexibility and regeneration capacity offered by successive cambia. Flexibility can be advantageous for trees and shrubs confronted with storms and/or heavy wind while a good regeneration capacity can be due to the presence of more than one cambium. Lastly, the increased storage of photosynthates is not only advantageous for lianas in their sprint to the canopy in order to receive light [2], but also for smaller trees growing in dense tropical forests.

### Conclusions and perspectives

**Figure 5:**
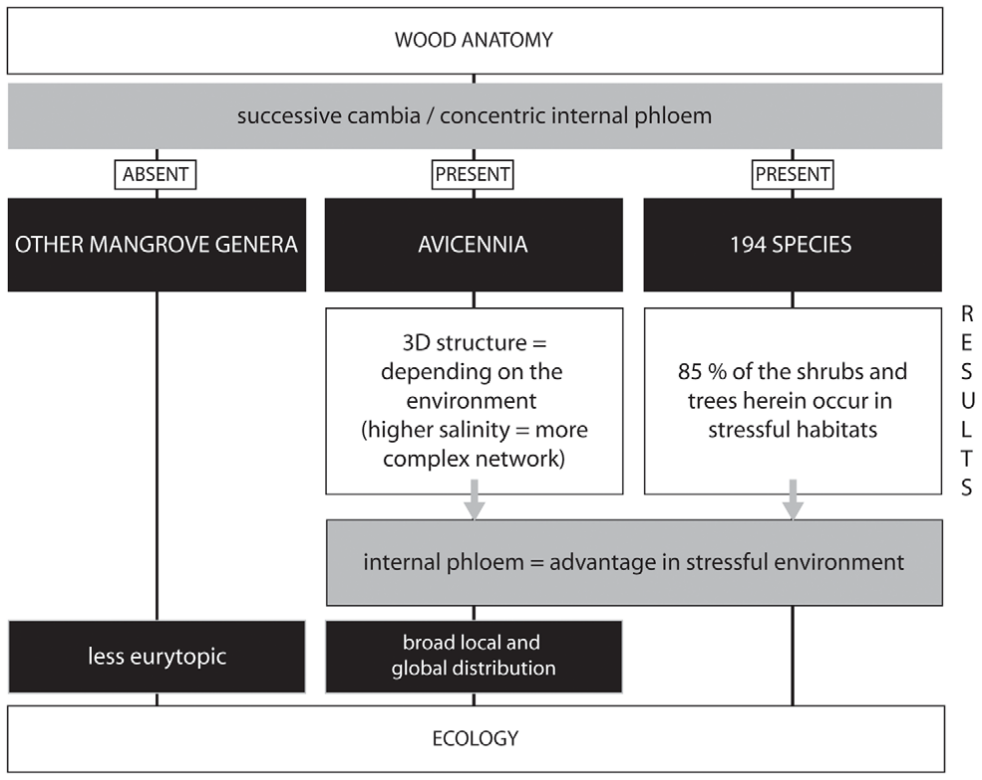
From wood anatomy to ecology: overview of the study. The flow chart is showing the organisms involved (black boxes, capital text) and the results of the study of successive cambia and concentric internal phloem in these organisms (white boxes, normal text) establishing that internal phloem could offer advantages in ecological stressful conditions (grey box with black frame). In this way, the wood anatomical features of *Avicennia* can help in explaining its broad distribution compared to other mangrove genera.

Growth via successive cambia offers an ecological advantage under water stress conditions. Woody species showing concentric internal phloem were predominant in salt or drought prone habitats, and the networking of the phloem tissue was more pronounced under harsher environmental conditions for the water transport (Fig. 5). Physiological experiments - *e.g.* those proving that the internal phloem tissue is still active [11] - should strengthen the hypothesis that the advantage is most probably related to the water storage capacity and the role in embolism repair of the internal phloem. Concentric internal phloem and thus growth via successive cambia was found to offer several ecological benefits for different groups of species growing in different habitats: under humid-wet conditions this anatomy offers ecological advantage for woody plants with a scandent growth, while under drought or salt stress conditions it can be beneficial for trees and shrubs. If this type of secondary growth is so beneficial, we can wonder why the commonly occurring growth through a single cambium is the main growth form of woody plants. The cost-benefit ratio of having more than one cambium might be the answer, although this has not been calculated to date, unless disadvantages as yet unknown to us would impede evolutionary spread of growth by more than one cambium.

The ecological benefits of successive cambia, in combination with the specialised vessel characteristics [39], help explaining the broad local and global distribution of *Avicennia* and its occurrence in extremely high salinity conditions. Generating data on the growth of species with network-like successive cambia at high temporal and spatial resolution with the laser-scanning method [40] or point dendrometers is needed to obtain more insight in the precise functioning of those cambia and the way the complex structure of the wood develops. The complexity of the water transport system structure that can originate from successive cambia is extending our current insight in plant secondary growth, as these types of structure can only be generated from cambia that are anatomically different from those generally observed in trees.

In conclusion, we can state that our study indicates that successive cambia are an important wood anatomical characteristic explaining ecological species distribution at least partially. It moreover stressed the importance of three-dimensional visualisations and interpretations of plant structures.

## Materials and Methods

### X-ray Computed Tomography (CT): CT-scanning

#### Wood Samples

In order to study the three-dimensional wood structure of the mangrove genus *Avicennia* L., we used (i) stems available in the xylarium of the Royal Museum of Central Africa in Tervuren (Belgium) and (ii) root samples and serial stem parts of one tree collected in the mangrove forest of Gazi Bay (Kenya).

Eighteen air-dried wood samples were selected from the Tervuren wood collection (Table 1). The samples were selected to allow comparison between (i) *Avicennia* species (*A. marina*, *A. officinalis* and *A. germinans*), (ii) biogeographical regions (eastern region: Indo-West Pacific and East Africa and western region: America and West Africa) and (iii) study sites mainly differing in degree of soil water salinity. All selected stems were at least 3 cm in diameter.

In addition to the stem samples out of the Tervuren wood collection, two cable root samples of *Avicennia marina* (Forssk.) Vierh. were collected in the mangrove forest of Gazi Bay, Kenya, in February 2009 at two different study sites (Table 1). Study of the variation in wood structure by height was done with twelve samples of the same tree (distance between samples: 50 cm) sampled from study site 2 of which a root sample also has been taken (Table 1). All collected samples were been air-dried before analysis and now are part of the Tervuren wood collection (Tw60819-20 – Table 1).

Environmental data from the different study sites in Gazi Bay, Kenya, were determined during former studies (Table 2) [3], [39].

#### Scanning Procedure

X-ray tomographical images have been made for all samples using a multi-slice spiral CT-scanner (CT-scan Brilliance 64 slice, Philips, The Netherlands) with the following characteristics: collimation: 20×0,625 mm; slice thickness: 0.7 mm; reconstructed slice interval: 0.35 mm; intensity: 330 mAs; tension: 140 kV; X-tube rotation time: 0.75 s; reconstruction filter: B-standard. The field of view and the pixel spacing were adapted to the object diameter, all with 512×512 image matrix. Radial and three-dimensional reconstructions were made on the Philips CT-scan with dedicated software.

#### Data Collection and Analysis

In order to investigate the degree of branching of the *Avicennia* vascular network, points of ending and branched phloem tissue (Fig. 2c–g) were counted on the CT-images using ImageJ 1.41k (Wayne Rasband, National Institute of Health, Bethesda, Maryland, USA). For every sample, measurements were made at three different heights at 30 mm distance. On every image the stem surface area was determined by the ellips tools in eFilm Lite 2.1.2 (Merge OEM, Mississauge, Canada) or after measuring the stem diameters in iQ-VIEW 2.5.0 (IMAGE Information Systems, London, UK). From these measurements we calculated (i) the degree of branching, defined as the sum of ending and branched phloem tissue *i.e.* the number of points were the growth segments are not concentric per surface area, giving an indication of the complexity of the network of transport tissue and (ii) the ratio of ending to branched phloem. On the same images the internal phloem surface area was determined semi-automatically (Color Range tool in Adobe Photoshop CS3 – version 10.0.1, Adobe Systems Inc., San Jose, California, USA). From these measurements, the xylem surface area (total stem surface area minus internal phloem surface area) as well as the ratio of internal phloem surface area to xylem surface area could be calculated.

Degree of branching, ratio of ending to branched phloem and ratio of phloem to xylem surface area were compared within and between species, biogeographical regions and study sites using Mann-Whitney U and Kruskal-Wallis tests. All statistical analyses were conducted using Statistica 7.0 (StatSoft Inc., Tulsa, OK, USA).

### X-ray Computed Tomography (CT): Micro-CT-scanning

The samples used for micro-CT-scanning were representative *A. marina* samples of Gazi Bay, Kenya, out of the Tervuren wood collection. The pictures showed in this article are from the Tw60821 *A. marina* sample.

A high resolution desktop X-ray micro-CT-system (SkyScan 1172, SkyScan, Belgium) with closed X-ray micro-focus source, was used for non-destructive visualization of the internal structure of *A*. *marina*. It was possible to obtain an isotropic pixel resolution of 7.5 µm using a camera binning mode of 2 by 2 pixels resulting in a projection image of 1000×2000 pixels. A filter of 0.5 mm aluminium was chosen in order to get a better contrast and to reduce the beam hardening effect. The peak voltage of the source was set at 90 kV. Projection images were taken with a rotation step of 0.4° over 180° and the signal to noise of the projection images was improved by using a frame averaging of 3. After acquiring the projection images the reconstruction was done using a modified Feldkamp cone-beam algorithm [41]. Two-dimensional cross-sectional images of the sample were obtained in consecutive slices throughout the object in order to obtain a three-dimensional dataset that can be viewed in any direction.

Three-dimensional smoothed surface images were made using the Reconstruct software (version 1.1.0.0 - John C. Fiala, Austin, Texas, USA) [42].

### Database Analysis

In order to upscale and verify the hypothesis that concentric internal phloem is an adaptive characteristic of species growing under drought stress, either by a lack of water or by the presence of salt, we conducted a database analysis in which we analysed 198 woody species with concentric internal phloem. Species were selected from the modern wood database of the Inside Wood Database [12] (species with IAWA (International Association of Wood Anatomists) characteristic 133: included phloem, concentric) or from scientific literature. For each species the growth habit, the habitat and the (bio)geography were searched for in the Inside Wood Database or in scientific articles and websites (Table S1). In all, 194 (87 genera out of 25 families) could been taken into the analysis (Table S1).

For statistical analysis (descriptive statistics) habitats were classified according to Walter's zonobiomes (Table S1) [43] based on both habitat and (bio)geography of each species. Zonobiomes II – IV and VII – IX have been associated with water stress as a result of drought during at least one period of the year and also saline habitats were considered physiologically dry. In contrast, zonobiomes I, V and VI as well as mountain areas were considered to be without extensive drought stress. Zonobiome II has been divided into II a (semi-evergreen and wet season green forests) and II b (savannas, grassland and dry woodlands) according to Walters's vegetational zones [43]. Habitat descriptions were decisive to divide species into stress categories (drought and/or salt stress versus no drought stress). Species with two growth habits (tree/shrub or shrub/liana) were assigned to both groups.

## Supporting Information

Table S1 **List of species with concentric internal phloem taken into account in the presented study.** Species names are according to the reference article while families are along the APG. Clear synonyms are removed from the list. For each species habit (L: liana, S: shrub or T: tree), habitat and (bio)geography have been searched for in scientific articles and websides. Based on this information, species have been categorised non salt tolerant (0) or at least salt tolerant (1) and have been classified to the different zonobiomes described in Walter's Vegetation of the Earth. Species from coastal areas have been classified as azonal (A), while species from mountain areas have been been indicted with mountain (M). Genera that were found to have internal concentric phloem are not taken into account in the analysis but only mentionend in this list. Number of species accoring to Mabberley's Plant-Book are mentioned between brackets.
(PDF)
