# Supplementary material for: Successive Cambia: A Developmental Oddity or an Adaptive Structure?
Source: PLoS One. 2011 Jan 31;6(1):e16558. doi: 10.1371/journal.pone.0016558 (PMC3031581; doi:10.1371/journal.pone.0016558)
Supplement: Table S1 — List of species with concentric internal phloem taken into account in the presented study. Species names are according to the reference article while families are along the APG. Clear synonyms are removed from the list. For each species habit (L: liana, S: shrub or T: tree), habitat and (bio)geography have been searched for in scientific articles and websides. Based on this information, species have been categorised non salt tolerant (0) or at least salt tolerant (1) and have been classified to the different zonobiomes described in Walter's Vegetation of the Earth. Species from coastal areas have been classified as azonal (A), while species from mountain areas have been been indicted with mountain (M). Genera that were found to have internal concentric phloem are not taken into account in the analysis but only mentionend in this list. Number of species accoring to Mabberley's Plant-Book are mentioned between brackets. (PDF) [file pone.0016558.s001.pdf]

Table S1: List of species with concentric internal phloem taken into account in the presented study. Species names are according to the reference article while families are along the APG [1]. Clear synonyms are removed from the list. For each species habit (L: liana, S: shrub or T: tree), habitat and (bio)geography have been searched for in scientific articles and websides. Based on this information, species have been categorised non salt tolerant (0) or at least salt tolerant (1) and have been classified to the different zonobiomes described in Walter's Vegetation of the Earth (see below table) [2]. Species from coastal areas have been classified as azonal (A), while species from mountain areas have been been indicted with mountain (M). Genera that were found to have internal concentric phloem are not taken into account in the analysis but only mentionend in this list. Number of species accoring to Mabberley's Plant-Book [3] are mentioned between brackets.

GENUS	SPECIES	FAMILY	HABIT	HABITAT	(BIO)GEOGRAPHY	SALT	WALTER	REFERENCE
Abuta	*brevifolia*	Menispermaceae	L	Rainforest	Middle and South America	0	I	[4-6]
Abuta	*colombiana*	Menispermaceae	L	Rainforest	Meso and South America	0	I	[4-6]
Abuta	*fluminum*	Menispermaceae	L	Amazonian forest	Peru, Amazonian area	0	I	[4-8]
Abuta	*grandiflolia*	Menispermaceae	L/S	Humid, tropical areas, near and far away from water, in secondary forest and degraded meadows	Tropical South America	0	I	[4, 5, 9]
Abuta	*grisebachii*	Menispermaceae	L	Rainforest	Meso and South America	0	I	[4-6, 10]
Abuta	*imene*	Menispermaceae	L	Rainforest	Brazil	0	I	[4, 5, 7, 11, 12]
Abuta	*rufescens*	Menispermaceae	L	Non-flooded moist forest	Tropical South America	0	I	[4, 5, 13]
Abuta	*sandwithiana*	Menispermaceae	L	Dense, submountainous ombrophilous forest	Brazil	0	I	[4, 5, 7, 14]
Albertisia	*exelliana*	Menispermaceae	L	Rainforest, near lake forest	Tropical Africa	0	I	[4, 15]
Albertisia	*papuana*	Menispermaceae	L	Rainforest	South East Asia	0	I	[4, 16]
Albertisia	*villosa*	Menispermaceae	L	Dense humid forest	Tropical Africa	0	I	[4, 17]
Allenrolfea	*vaginata*	Amaranthaceae	T	Desert with saline soils	Temperate South America including Argentina, Chile, Uruguay and South Paraguay	1	III-VII	[5, 18]
Anabasis	*articulata*	Amaranthaceae	S	Shrub steppe, desert	Mediterranean including Northern Africa and Middle East	1	III-IV	[5, 19]
Anamirta	*cocculus*	Menispermaceae	L/S	Tropical evergreen forest	India, Indonesia, Sri-Lanka	0	I	[4, 5, 20, 21]
Anisocycla	*cymosa*	Menispermaceae	L	Sandy riversides and fringing forest	Congo	0	I	[4, 22]
Anomospermum	*chloranthum*	Menispermaceae	L	Moist forest	Meso and South America	0	I	[4, 5, 23]
Anomospermum	*reticulatum*	Menispermaceae	L	Dense tropical evergreen forest	Costa Rica, Southern America	0	I	[4, 24, 25]
Arcangelisia	*flava*	Menispermaceae	L	Wet forest, near river banks	Temperate China, tropical Asia	0	I-II-V-VI	[3, 4, 25]

GENUS	SPECIES	FAMILY	HABIT	HABITAT	(BIO)GEOGRAPHY	SALT	WALTER	REFERENCE
Arthraerua	*leubnitziae*	Amaranthaceae	S	Desert and sand dunes	Namibia	0	III	[26]
Arthrocnemum	*macrostachyum*	Amaranthaceae	S	Saline habitats: coasts and banks of saline water pools	Mediterranean including Northern Africa and Middle East	1	IV	[5, 27]
Arthrocnemum	*perenne*	Amaranthaceae	S	Muddy salt marshes near the sea	Mediterranean including Northern Africa and Middle East	1	IV	[5, 15]
Atriplex	*halimus*	Amaranthaceae	S	Dry and saline habitats, desert	Mediterranean including Northern Africa and Middle East	1	IV	[5, 28]
Atriplex	*nummularia*	Amaranthaceae	S	Arid and semi arid regions, usually on saline soils	Australia	1	III	[29, 30]
Avicennia	*alba*	Acanthaceae	S/T	Mangrove forests	Indo-Pacific ocean	1	A	[31-34]
Avicennia	*bicolor*	Acanthaceae	S/T	Mangrove forests	West coast of Meso America	1	A	[31-34]
Avicennia	*germinans*	Acanthaceae	S/T	Mangrove forests	Meso America (up to Florida) and West Afica	1	A	[5, 31-37]
Avicennia	*integra*	Acanthaceae	S/T	Mangrove forests	North Australia	1	A	[31, 33, 34, 38]
Avicennia	*marina*	Acanthaceae	S/T	Mangrove forests	East Africa and Indo-Pacific	1	A	[5, 31-34, 38-40]
Avicennia	*officinalis*	Acanthaceae	S/T	Mangrove forests	Indo-Pacific	1	A	[31-34, 41]
Avicennia	*rumphiana*	Acanthaceae	S/T	Mangrove forests	Indo-Malaysia	1	A	[31, 33, 34]
Avicennia	*schauriana*	Acanthaceae	S/T	Mangrove forests	East coast of South America	1	A	[31-34]
Azima	*tetracantha*	Salvadoraceae	S	Tropical mainland, on saline or alluvial soils near rivers and coast	Tropical mainland Africa and adjacent islands (Madagascar, Mauritius, Réunion, and Comores)	1	I-IIa-IIb-III	[5, 15, 42]
Barbeuia	*madagascariensis*	Barbeuiaceae	L	Subhumid to humid forest	Madagascar	0	I-II a	[43-45]
Beirnaertia	*cabindensis*	Menispermaceae	L	Tropical wet forest	Tropical Africa	0	I	[3, 4, 46]
Bosea	*yervamora*	Amaranthaceae	S	Dry areas	Madeira and the Canary Islands	0	IV	[5, 26]
Bougainvillea	*spectabilis*	Nyctaginaceae	L/S	Rainforest	Brazil, naturalised elsewhere	0	I	[5, 25, 47-50]
Bredemeyera	sp.	Polygalaceae	S	Caatinga, forest patches in a savannah biome	Neotropics and temperate Brazil, tropical South American, New Guinea, tropical Australia	0	II b	[3, 5, 51]
Cadaba	*glandulosa*	Capparaceae	S	Deciduous bush land and grassland, dry places on clay or sandy soil and dry riverbeds	Mediterranean including Northern Africa and Middle East, Tropical mainland Africa and adjacent islands (Madagascar, Mauritius, Réunion, and Comores)	1	II b - IV	[5, 27]
Cadaba	*rotundifolia*	Capparaceae	S	Deciduous bush land, semi-desert and dry riverbeds	Mediterranean including Northern Africa and Middle East, tropical	1	II b – IV	[5, 27]

GENUS	SPECIES	FAMILY	HABIT	HABITAT	(BIO)GEOGRAPHY	SALT	WALTER	REFERENCE
					Africa and adjacent islands			
Caryomene	*olivascens*	Menispermaceae	L	Tropical rainforest	Meso and South America	0	I	[4, 5, 52]
Casimirella	*ampla*	Icacinaceae	L	Moist deciduous and semi-evergreen seasonal forest, near riverbanks	Tropical South America	0	II a	[5, 53]
Celosia	*floribunda*	Amaranthaceae	T	Dry land, scrubland	Mexico (Baja California)	0	III	[26]
Ceratoides	*lanata*	Amaranthaceae	S	Steppe and grassland, on saline, clay or calcareous soils	North America, north of Mexico	1	VII	[5, 54]
Charpentiera	*densiflora*	Amaranthaceae	T	Mesic forest	Hawaiian Islands	0	II a	[26, 55]
Charpentiera	*elliptica*	Amaranthaceae	T	Lowland wet or mesic forest	Hawaiian Islands	0	II a	[26, 56]
Charpentiera	*obovata*	Amaranthaceae	S	Mesic to occasionally wet rain forests	Pacific Islands (including New Caledonia, Samoa, Hawaii and Fiji)	0	II a	[5, 26]
Cheiloclinium	spp. (11)	Celastraceae	L	Campinarana forest	Mexico and Central America, tropical South America	0	II	[5, 57]
Cheiloclinium	*anomalum*	Celastraceae	L	Rainforest	Amazonian area	0	I	[5, 7, 58, 59]
Cheiloclinium	*belizense*	Celastraceae	L	Riparian forest	Belize, Panama, Brazil, Venezuela	0	I – II	[5, 13, 58, 60]
Cheiloclinium	*hippocrateoides*	Celastraceae	L	Lowland tropical to subtropical moist forest	South America	0	II a	[5, 13, 58, 61]
Cheiloclinium	*serratum*	Celastraceae	L	Riverine habitats	Middle and South America	0	I-II-III-V-VII	[5, 58, 62, 63]
Chenopodium	*sandwicheum*	Amaranthaceae	S	Dry land habitats from coastal to subalpine, adapted to drought	Pacific Islands (including New Caledonia, Samoa, Hawaii, and Fiji)	1	II b	[5, 64]
Chondrodendron	*microphyllum*	Menispermaceae	L	Rainforest	Meso and South America	0	I	[4, 65]
Chondrodendron	*tomentosum*	Menispermaceae	L	Rainforest	Meso and South America	0	I	[4, 65]
Cissampelos	*pareira*	Menispermaceae	L/S	Riverine and lowland forest	Tropical regions worldwide	0	I	[4, 5, 17, 49]
Cocculus	*hirsutus*	Menispermaceae	L/S	Lowland and medium-altitude bush land and woodland	India, Pakistan, Tropical Africa	0	II a - II b	[45, 47, 66, 67]
Cocculus	*laurifolius*	Menispermaceae	L	Mountain region, dry evergreen forest between 1200 and 1600 m	Temperate Asia (China, Japan and Russia)	0	M	[4, 5, 68]
Cocculus	*pendulus*	Menispermaceae	L	Savannah	Tropical and subtropical India, Pakistan and Africa	0	IIb	[4, 5, 17, 45, 69]
Colignonia	*scandens*	Nyctaginaceae	S	Arid regions, mountains > 3000 m	Tropical South America	0	M	[5, 70]
Combretum	*nigricans*	Combretaceae	S/T	Savannah	Tropical Africa and adjacent islands	0	II b	[5, 71]

GENUS	SPECIES	FAMILY	HABIT	HABITAT	(BIO)GEOGRAPHY	SALT	WALTER	REFERENCE
Curarea	*candicans*	Menispermaceae	L	Tropical lowland forest	Neotropics	0	I	[4, 5, 72]
Curarea	*tecunarum*	Menispermaceae	L	Tropical lowland forest	Neotropics	0	I	[4, 5, 72]
Curarea	*toxicofera*	Menispermaceae	L	Tropical lowland forest	Neotropics	0	I	[4, 5, 72]
Cycas	*circinalis*	Cycadaceae	T	Along the seashore, inland and occasionally in mountainous areas, open grass lands, shrub-woodlands in hilly areas to dense forests	India, South-East Asia	1	II a - II b – (M)	[73-77]
Cycas	*rumphii*	Cycadaceae	T	Tropical woodland or forest, mainly along coastal areas, generally on sandy soil and calcareous substrate	Moluccan island group, Papua, Indonesia	1	II a	[78-81]
Cycas	*thuarsii*	Cycadaceae	T	Near coast, behind the dunes, in open bush land	Madagascar, coast of East Africa	1	II a - II b	[15, 78, 81]
Dalbergia	*lanceolaria*	Fabaceae	T	Tropical dry thorn forest, tropical moist deciduous forest	Central South Asia	0	II a - II b	[5, 73, 82]
Dalbergia	*paniculata*	Fabaceae	T	Primary moist deciduous forest, mesomorphic habitat	India, Indo-China (tropical Asia)	0	II a	[25, 83]
Dicranostyles	*guianensis*	Convolvulaceae	L	Evergreen broadleaf rainforest with high rainfall	Tropical South America	0	I	[5, 53]
Dicranostyles	*mildbraediana*	Convolvulaceae	L	Evergreen broadleaf rainforest with high rainfall	Tropical South America	0	I	[5, 53]
Dicranostyles	*villosus*	Convolvulaceae	L	Evergreen broadleaf rainforest with high rainfall	Mexico and Central America	0	I	[5, 53]
Dioscoreophyllum	*cumminsii*	Menispermaceae	L	Evergreen tropical rainforest	Tropical Africa	0	I	[4, 72, 84]
Diploclisia	*glaucescens*	Menispermaceae	L	Tropical rainforest	Tropical Asia	0	I	[4, 45]
Doliocarpus	spp. (*ca.* 40)	Dilleniaceae	L	Transition gallery forest, cerrado	Mexico and Central America, tropical South America	0	I-II a	[5, 57]
Doliocarpus	*coriaceus*	Dilleniaceae	L	Wet lowland forest	Mexico and Central America, tropical South America	0	I	[5, 85]
Elephantomene	*eburnea*	Menispermaceae	L	Tropical rainforest	Meso and South America	0	I	[4, 5, 53]
**Forchhammeria*	*longifolia*	Capparaceae	S		Mexico and Central America	0		[5, 86]
Gallesia	*integrifolia*	Phytolaccaceae	T	Semi-deciduous forest, upland forest with dense closed canopy, tropical lowland dry deciduous forest	Tropical South America	0	II a	[5, 87-89]
Gallesia	*scorododendrum*	Phytolaccaceae	T	Atlantic moist forest	Temperate South America including	0	I	[5, 90]

GENUS	SPECIES	FAMILY	HABIT	HABITAT	(BIO)GEOGRAPHY	SALT	WALTER	REFERENCE
					Argentina, Chile, Uruguay and South Paraguay			
Gnetum	*ula*	Gnetaceae	L	Moist deciduous rainforest	India (Eastern and Western Ghats)	0	II a	[47, 73, 91-95]
Haematocarpus	*subpeltatus*	Menispermaceae	L	Tropical forest along streams at low altitudes	Southeast Asia	0	II a	[4, 96]
Haloxylon	*persicum*	Amaranthaceae	S/T	Sand hills, deserts and sand ridges	Mediterranean including Northern Africa and Middle East	1	III-IV-VI-VII-VIII	[5, 97]
Haloxylon	*salicornicum*	Amaranthaceae	S	Desert and semi-desert areas in soils containing much salt	Northern Africa, Arabian peninsula, Western Asia, Indian subcontinent	1	III - IV – VII	[25, 98]
Hyperbaena	*domingensis*	Menispermaceae	L	Seasonal humid Amazonian rainforest, flooded grassland forest	Tropical South America	0	I - II a	[4, 5, 99]
Hyperbaena	*winzerlingii*	Menispermaceae	L	Flooded grassland forests	Tropical South America	0	I - II a	[5, 100]
Hypserpa	*nitida*	Menispermaceae	L	Tropical rainforest, forest margins	Southeast Asia	0	I	[4, 5, 101]
Ipomoea	*arborescens*	Convolvulaceae	T	Dry tropical forest	Mexico (Sonora)	0	II a	[102, 103]
Ipomoea	*murucoides*	Convolvulaceae	L	Tropical deciduous moist forest	Mexico, South America, tropical Africa and Southeast Asia. Australia	0	II a	[5, 102]
Ipomoea	*pauciflora*	Convolvulaceae	T	Dry coastal forests	Central Mexico, Meso America, Western South America	0	II a – III – IV	[25, 102, 104]
Ipomoea	*pentaphylla*	Convolvulaceae	L	Tropical beach, sand dunes, in forests on calcareous rock	Circumtropical	1	A	[47, 59]
Ipomoea	*pes-caprae*	Convolvulaceae	L	Sand dunes and beaches	Circumtropical	1	A	[47, 105]
Ipomoea	*praecana*	Convolvulaceae	L	Dry thicket or forest	Southern Mexico, Honduras, Nicaragua	0	II a	[85, 92, 102]
Ipomoea	*populina*	Convolvulaceae	L	Xeric shrub land	Mexico, Meso America	0	II a	[102, 106, 107]
Ipomoea	*wolcottiana*	Convolvulaceae	T	Tropical dry forest	Pacific slope of southern Mexico / Mexico (Northern America)	0	II a	[5, 86, 102, 103, 108]
Iresine	sp.	Amaranthaceae	S	Subtropical or tropical dry forest	Tropical South America, temperate South America including Argentina, Chile, Uruguay and South Paraguay, West Africa, South Japan	0	II b	[3, 5, 92]
Kochia	*sedifolia*	Amaranthaceae	S	Dry, open shrub land	Australia	1	II b –III – IV	[29, 98]
Koompassia	*malaccensis*	Fabaceae	T	Mixed swamp forest, Kerangas forest, moist deciduous and semi-evergreen seasonal forest	Indochina and Indomalaysia	0	II a	[5, 109]

GENUS	SPECIES	FAMILY	HABIT	HABITAT	(BIO)GEOGRAPHY	SALT	WALTER	REFERENCE
**Legnephora*	*minutiflora*	Menispermaceae	L		Asia	0		[4]
Machaerium	*cobanense*	Fabaceae	L	Humid forest	Neotropics	0	I	[110-112]
Machaerium	*floribundum*	Fabaceae	L	Wet tropical rainforest	Mesoamerica	0	I	[5, 110, 113]
Macrococculus	*pomiferus*	Menispermaceae	L	Tropical rainforest	Southeast Asia (Indonesia)	0	I	[4, 53]
Maerua	*angolensis*	Capparaceae	S/T	Very dry areas, savannah	Tropical mainland Africa and adjacent islands (Madagascar, Mauritius, Réunion, and Comores), Southern Africa (south of the Tropic of Capricorn)	0	II b, III, IV	[5, 15]
Maerua	*filliformis*	Capparaceae	T	Very dry areas, savannah	Madagascar	0	II b	[5, 15, 45]
Maerua	*oblonfifolia*	Capparaceae	S	Savannah woodland	Mediterranean including Northern Africa and Middle East, tropical Africa and adjacent islands	0	II b, III, IV	[5, 15]
Maerua	*rigida*	Capparaceae	S/T	Very dry areas, savannah	Tropical mainland Africa and adjacent islands (Madagascar, Mauritius, Réunion, and Comores), Southern Africa (south of the Tropic of Capricorn)	0	II b	[5, 15]
Maerua	*rosmarinoides*	Capparaceae	S/T	Woodland and bush land	Southern Africa (south of the Tropic of Capricorn)	0	II b	[5, 114]
Mestoklema	*tuberosum*	Aizoaceae	S	Shrub land	Cape (South Africa)	0	IV	[3, 112, 115]
Nototrichum	*sandwicense*	Amaranthaceae	S	Open dry forest	Hawaiian Islands	0	II a - II b	[26, 116]
Orthomene	*schomburgkii*	Menispermaceae	L	Riparian rainforest	Meso and South America	0	I	[4, 117]
Pachygone	*dasycarpa*	Menispermaceae	L	Rainforest	Southeast Asia	0	I	[4, 118]
Pera	*bicolor*	Euphorbiaceae	T	Moist deciduous and semi-evergreen seasonal forest	Tropical South America	0	II a	[5, 53]
Peritassa	spp. (14)	Celastraceae	L/S	Lowland, non-flooded, tropical rainforest	South America, Tobago	0	I	[5, 7, 53]
Peritassa	*bullata*	Celastraceae	L	Tropical forest	Ecuador	0	I	[5, 58, 119, 120]
Peritassa	*calypsoides*	Celastraceae	S	Non-flooded open bush	South Brazil	0	II a	[5, 58, 121, 122]
Peritassa	*huanucana*	Celastraceae	L	Riparian forest	Range includes Peru and Suriname	0	I (II b - III)	[5, 13, 58, 60]
Peritassa	*pruinosa*	Celastraceae	L	Dense and high Terra Firme lowland forest at low altitudes	Meso-America, northern South America	0	I	[13, 58, 123]

GENUS	SPECIES	FAMILY	HABIT	HABITAT	(BIO)GEOGRAPHY	SALT	WALTER	REFERENCE
Petiveria	*alliacea*	Phytolaccaceae	S	Wet forest	Tropical Africa, South America and Mesoamerica, Caribbean	0	I-II a	[87, 92, 124-126]
Pfaffia	*grandiflora*	Amaranthaceae	S	Lowland tropical to subtropical moist forest, Terra Firme forest	Temperate South America including Argentina, Chile, Uruguay and South Paraguay	0	II a	[5, 61]
Phytolacca	*dioica*	Phytolaccaceae	T	Pampas grassland	Brazil, Peru, Paraguay, Uruguay, North of Argentina	1	II b - VII	[50, 87, 127]
Phytolacca	*dodecandra*	Phytolaccaceae	L	Wide range of habitats, often riverine, evergreen bush land	Tropical Africa, Madagascar	0	II a	[87, 128, 129]
Phytolacca	*weberbaueri*	Phytolaccaceae	T	Pampas grassland	Brazil, Peru, Paraguay, Uruguay, North of Argentina	0	II b – VII	[50, 127, 130]
Pisonia	*brunoniana*	Nyctaginaceae	S/T	Dry to mesic forest, gulches and occasionally wet areas, especially along the sides of streams	Southeast Asia and Pacific islands, Australia and New Zealand	0	II b	[5, 48, 131, 132]
Polygala	sp.	Polygalaceae	S	Coastal or inland habitats in xeric vegetation, from dry to moist habitats	Subcosmopolitan (not in New Zealand)	1	IIa-IIb -III	[3, 5, 133, 134]
Pycnarrhena	*cauliflora*	Menispermaceae	L	Tropical rainforest	Southeast Asia	0	I	[4, 53]
Pycnarrhena	*celebica*	Menispermaceae	L	Tropical rainforest	Southeast Asia	0	I	[4, 53]
Pycnarrhena	*lucida*	Menispermaceae	L	Tropical forest	Southeast Asia	0	I	[4, 53]
Pycnarrhena	*tumefacta*	Menispermaceae	L	Tropical rainforest	Southeast Asia	0	I	[4, 45]
Rhabdodendron	*amazonicum*	Rhabdodendraceae	T	Riparian forest, lowland forest, Terra Firme forest	Tropical South America	0	I	[5, 13, 57, 135]
Salacia	spp. (200)	Celastraceae	L/S/T	Subtropical or tropical moist lowland rainforest	Africa, Madagascar, Malaysia, neotropics	0	I	[5, 24, 92]
Salacia	*adolfo-friderici*	Celastraceae	L	Tropical moist semi-decideous forest	Cameroon, Ghana	0	I - II a	[5, 58, 86, 136]
Salacia	*alwynii*	Celastraceae	L	Rainforest	Western South America	0	I	[5, 58, 86, 137, 138]
Salacia	*amplectens*	Celastraceae	L	Dense and high Terra Firme lowland forest at low altitudes	Brazil, …	0	I	[5, 13, 58, 86]
Salacia	*cauliflora*	Celastraceae	L	Rainforest	Amazonian area	0	I	[53, 58, 86, 139]
Salacia	*cerasifera*	Celastraceae	L	Guineo-congololese forest	Tropical Africa	0	I	[5, 45, 58, 86]
Salacia	*chinensis*	Celastraceae	L/S	Hot and humid forest	Africa, Indochina, Australia	0	I	[45, 58, 140]
Salacia	*chlorantha*	Celastraceae	L/S	Evergreen forest and fringing	Tropical Africa	0	I	[15, 58, 86]

GENUS	SPECIES	FAMILY	HABIT	HABITAT	(BIO)GEOGRAPHY	SALT	WALTER	REFERENCE
				forest				
Salacia	*crassifolia*	Celastraceae	T	Cerrado	Southern America	0	II b	[5, 58, 86, 141]
Salacia	*debilis*	Celastraceae	L	Tropical forest	West Tropical Africa	0	I	[5, 15, 58, 86]
Salacia	*disepala*	Celastraceae	L	Rainforest	Australia, …	0	II b	[58, 142]
Salacia	*duckei*	Celastraceae	L	Tropical rainforest	Southern America	0	I	[5, 58, 86, 143]
Salacia	*elegans*	Celastraceae	L/S	Evergreen and fringing forest and dense deciduous woodland	Tropical Africa	0	I-II a	[5, 15, 58, 86]
Salacia	*erecta*	Celastraceae	L/S	Evergreen forest or thicket, 50-1700 m	Tropical Africa	0	I	[58, 86, 144]
Salacia	*germainii*	Celastraceae	L	African rainforest	Congo	0	I	[5, 27, 53, 58, 86]
Salacia	*impressifolia*	Celastraceae	L	Neotropical rainforest	Central and South America	0	I	[5, 58, 145, 146]
Salacia	*juruana*	Celastraceae	L	Lowland tropical to subtropical moist forest	Amazonian area	0	I	[5, 58, 61, 86]
Salacia	*kanukuensis*	Celastraceae	L	Dense tropical forest	Northern South America, Southern America	0	I	[5, 58, 86, 139, 147]
Salacia	*lateritia*	Celastraceae	L	Tropical rainforest	Tropical Africa	0	I	[15, 53, 58, 86]
Salacia	*letestui*	Celastraceae	L	West African rainforest	Congo	0	I	[5, 58, 86, 148]
Salacia	*macrantha*	Celastraceae	T	Lowland tropical to subtropical moist forest	Amazonian area	0	I	[5, 58, 61, 86, 149]
**Salacia*	*miqueliana*	Celastraceae	L	Riparian forest		0		[13, 58, 86]
Salacia	*multiflora*	Celastraceae	L	Dense, humid evergreen forest		0	I	[5, 13, 58, 86, 145]
Salacia	*nitida*	Celastraceae	L	Tropical forest	Tropical Africa	0	I	[15, 58, 86]
Salacia	*opacifolia*	Celastraceae	L	Submontaneous dense rainforest	Neotropics	0	I	[24, 58, 150]
Salacia	*prinoides*	Celastraceae	L	Forest along seashore, riverbanks	India, …	1	II a - II b	[5, 58, 151]
Salacia	*pynaertii*	Celastraceae	L	Evergreen and fringing forest	Tropical Africa	0	I	[5, 58, 86, 144]
Salacia	*reticulata*	Celastraceae	L	Subtropical or tropical moist lowland rainforest	Central South Asia (India, Pakistan, Sri Lanka)	0	I	[5, 58, 92]
Salacia	*solimoesensis*	Celastraceae	L	Old growth tropical to subtropical moist forest	Amazonian area	0	I	[5, 58, 61, 86]
Salacia	*staudtiana*	Celastraceae	L	Tropical rainforest	Tropical Africa	0	I	[5, 15, 58, 86]

GENUS	SPECIES	FAMILY	HABIT	HABITAT	(BIO)GEOGRAPHY	SALT	WALTER	REFERENCE
Salacia	*whytei*	Celastraceae	L	Rainforest	Tropical Africa	0	I	[15, 58, 86]
Salsola	*baryosma*	Amaranthaceae	S	Saline and waste sandy places, Sahara desert	Mediterranean including Northern Africa and Middle East	1	III-IV	[5, 15]
Salvadora	*persica*	Salvadoraceae	S/T	Thorn shrub land, grassy savannahs, coastal or riverine scrub on saline, sandy or alluvial soils, tropical semi-arid areas	Central South Asia, Mediterranean including Northern Africa and Middle East, Tropical Africa and adjacent islands	1	II b – III – IV - VII	[5, 15, 42, 152]
Sarcobatus	*vermiculatus*	Sarcobataceae	S	Strongly alkaline and saline soils, in semi-arid to arid plains, in badlands, silt dunes	North America, north of Mexico	1	III	[5, 153, 154]
Sciadotenia	*eichleriana*	Menispermaceae	L	Moist forest	Meso and South America	0	I	[4, 23]
Sciadotenia	*sprucei*	Menispermaceae	L	Neotropical rainforest	Meso and South America	0	I	[4, 5, 155]
Sciadotenia	*toxicofera*	Menispermaceae	L	Floodplain, Terra Firme forest	Meso and South America	0	I	[4, 5, 156]
Securidaca	*diversifolia*	Polygalaceae	T	Tropical dry to premontaneous wet forest	Mexico and Central America, tropical South America	0	II a	[5, 157]
Securidaca	*philippinensis*	Polygalaceae	L	Rainforest	Indomalaysia	0	I	[5, 158]
Securidaca	*virgata*	Polygalaceae	L/S	Subtropical wet and moist valley forest and hillsides	Caribbean	0	I-II a	[5, 159]
Seguieria	*americana*	Phytolaccaceae	S	Subtropical seasonal forest	South America	0	II a	[25, 87, 160, 161]
Seguieria	*paraguayensis*	Phytolaccaceae	T	Semi-evergreen seasonal forest, tropical moist forest, deciduous forest	Temperate South America including Argentina, Chile, Uruguay and South Paraguay	0	II a – IV	[5, 53, 162]
Simmondsia	*chinensis*	Simmondsiaceae	S	Desert and chaparral	Sonoran and Mojave Desert (southwest US, northwest Mexico)	1	III	[163-165]
Sleumeria	*auriculata*	Icacinaceae	L	Rainforest	North Borneo	0	I	[3, 166]
Solenostemma	*argel*	Apocynaceae	S	Dry sandy semi-desert areas	Mediterranean including Northern Africa and Middle East, Sahara (Arabia)	1	III	[5, 15]
Spatholobus	*roxburghii*	Fabaceae	L	Sal and teak forest, tropical forest with high rainfall	southeast Asia	0	II a	[167]
Stegnosperma	*cubense*	Phytolaccaceae	L/S	Tropical dry forest	Cuba, Dominican Republic, Jamaica, Pacific Coast and central Mexico	0	II a	[24, 168-171]
Stegnosperma	*halimifolium*	Phytolaccaceae	S	Beach, along permanent and ephemeral watercourses, to fifty miles from the coast	coastal Baja California, Mexico	1	A	[168, 169, 171, 172]

GENUS	SPECIES	FAMILY	HABIT	HABITAT	(BIO)GEOGRAPHY	SALT	WALTER	REFERENCE
Stegnosperma	*watsonii*	Phytolaccaceae	S	Along seacoast and about saline swales	Mexico, coastal Sonora, northern Sinaloa	1	A	[168, 169, 171, 173]
Strychnos	*glabra*	Loganiaceae	T	Semi-evergreen seasonal forest, tropical moist forest	Tropical South America	0	II a	[5, 53]
Suaeda	*fruticosa*	Amaranthaceae	S	Saline alluvial flats with clayey or sandy soils, on drier sites and coastal belts	Mediterranean including Northern Africa and Middle East	1	A	[5, 45]
Suaeda	*monoica*	Amaranthaceae	S	Coastal habitat, halophytic, able to tolerate frequent sea-water flooding	East Africa and Asia	1	A	[5, 15]
Syrrheonema	*fasciculatum*	Menispermaceae	L	Rainforest, forest regrowth and fallow land, at 1200–1400 m altitude	Tropical Africa	0	I -II a – M	[4, 156]
Telitoxicum	*glaziovii*	Menispermaceae	L	Tropical lowland rainforest	Meso and South America	0	I	[4, 52]
Telitoxicum	*krukovii*	Menispermaceae	L	Neotropical rainforest	Meso and South America	0	I	[4, 155]
Telitoxicum	*minutiflorum*	Menispermaceae	L	Neotropical rainforest	Meso and South America	0	I	[4, 155]
Telitoxicum	*peruvianum*	Menispermaceae	L	Rainforest	Meso and South America	0	I	[4, 155]
Tetracera	*volubilis*	Dilleniaceae	L	Shrubby forest, semi-deciduous tropical dry forest	Mexico and Central America, tropical South America	0	II b	[5, 126]
Tetrastigma	*voinierianum*	Vitaceae	L	Wet rainforest	Indo-China (Vietnam and Laos)	0	I	[5, 174-176]
Tiliacora	*acuminata*	Menispermaceae	L	Riverine, deciduous forests	China, Nepal, Ceylon, Burma	0	II a	[4, 45, 177]
Tiliacora	*chrysobotrya*	Menispermaceae	L	Rainforest	Angola, Congo	0	I	[4, 53, 178]
Tiliacora	*dielsiana*	Menispermaceae	L	Rainforest and fringing forest	Guinea, Liberia, Ivory Coast, Ghana	0	I	[4, 22]
Tiliacora	*funifera*	Menispermaceae	L	In riverine and other evergreen forest and moist areas in woodland	Tropical Africa	0	I -II a	[4, 5, 179]
Tiliacora	*glycosmantha*	Menispermaceae	L	Lowland and upland rain-forest, riverine forest and moist places in woodland	Tropical mainland Africa and adjacent islands (Madagascar, Mauritius, Réunion, and Comores)	0	II a	[5, 15]
Tiliacora	*laurentii*	Menispermaceae	L	Forest, subtropical and tropical moist lowland	Tropical Africa	0	II a	[4, 180]
Tontelea	*corymbosa*	Celastraceae	L	Neotropical rainforest	Amazonian area	0	I	[5, 7, 58, 155]
Tontelea	*mauritioides*	Celastraceae	L	Forest on Terra Firme	Brazil	0	I	[5, 7, 58, 181]
Tontelea	*micrantha*	Celastraceae	S/T	Dry grassland	Brazil	0	II b	[5, 7, 58, 182]
Tontelea	*nectandrifolia*	Celastraceae	L	Slope forest with well drained soil	South America	0	II a	[5, 7, 13, 58]

GENUS	SPECIES	FAMILY	HABIT	HABITAT	(BIO)GEOGRAPHY	SALT	WALTER	REFERENCE
Triclisia	*dictyophylla*	Menispermaceae	L/S	Dense, humid lowland to medium-altitude forest	Tropical Africa	0	I – II a	[4, 22]
**Triclisia*	*jumelleana*	Menispermaceae	L		Africa and Madagascar	0		[4, 183]
Triclisia	*patens*	Menispermaceae	L	Lowland rainforest	Tropical West Africa	0	I	[4, 15]
Triclisia	*sacleuxii*	Menispermaceae	L	Lowland rainforest and riverine forest	Tropical Africa	0	I	[4, 15]
Wisteria	*floribunda*	Fabaceae	L	Forest edges and disturbed areas at low altitudes, can tolerate a variety of soil and moisture types but it prefers loamy, deep, well drained soils	Temperate Asia (China), Japan and Russia	0	V	[5, 184]

* No appropriate data could be found so that the species has not been included in the analysis of the data.

Zonobiomes according to Breckle 2002 [2]:

I Zonobiome of the Evergreen Tropical Rain Forest (Zonobiome of the Equatorial Humid Diurnal Climate)
II Zonobiome of Savannas and Deciduous Forests and Grasslands (Zonobiome of the Humido-arid Tropical Summer Rain Region)
II a semi-evergreen and wet season green forests
II b savannas, grassland and dry woodlands
III Zonobiome of Hot Deserts (Zonobiome of Subtropical Arid Climates)
IV Zonobiome of Sclerophyllic Woodlands (Zonobiome of the Arido-Humd Winter Rain Region)
V Zonobiome of Laurel Forests (Zonobiome of the Warm-Temperate Humid Climate)
VI Zonobiome of Decideous Forests (Zonobiome of the Temperate Nemoral Climate)
VII Zonobiome of Steppes and Cold Deserts (Zonobiome of the Arid-Temperature Climate)
VIII Zonobiome of the Taiga (Zonobiome of the Cold-Temperature Boreal Climate)
IX Zonobiome of the Tundra (Zonobiome of the Arctic Climate)

References:

1. Angiosperm Phylogeny Website. Version 9, June 2008 [and more or less continuously updated since] - http://www.mobot.org/MOBOT/research/APweb/ - [Date accessed: 26th of January 2010]
2. Breckle, S.-W. (2002). Walter's Vegetation of the Earth. The Ecological Systems of the Geo-Biosphere. Berlin - Heidelberg - New York: Springer-Verlag.
3. Mabberley, D.J. (2008). Mabberley's Plant-Book: A Portable Dictionary of Plants, Their Classification and Uses Book Description. Cambridge: Cambridge University Press.
4. Jacques, F.M.B., and De Franceschi, D. (2007). Menispermaceae wood anatomy and cambial variants. Iawa Journal *28*, 139-172.
5. Inside Wood Database. http://insidewood.lib.ncsu.edu/search - [Date accessed: 26th of January 2010]
6. Krukoff, B.A., and Barneby, R.C. (1973). Supplementary notes on American Menispermaceae. Phytologia *25* - Accessed from the World Wide Web on the 26th of January 2010: http://www.archive.org/stream/phytologia36glea/phytologia36glea_djvu.txt

7. The Wood Explorer - http://www.thewoodexplorer.com/ - [Date accessed: 26th of January 2010]
8. Culot, L., Huynen, M.C., Gerard, P., and Heymann, E.W. (2009). Short-term post-dispersal fate of seeds defecated by two small primate species (*Saguinus mystax* and *Saguinus fuscicollis*) in the Amazonian forest of Peru. Journal of Tropical Ecology *25*, 229-238.
9. Maya Ethnobotanicals - http://www.maya-ethnobotanicals.com/ - [Date accessed: 26th of January 2010]
10. Vamosi, S.M., Mazer, S.J., and Cornejo, F. (2008). Breeding systems and seed size in a Neotropical flora: Testing evolutionary hypotheses. Ecology *89*, 2461-2472.
11. Silverton, J.V., Kabuto, C., Buck, K.T., and Cava, M.P. (1977). Structure of Imerubrine, a Novel Condensed Tropolone-Isoquinoline Alkaloid. Journal of the American Chemical Society *99*, 6708-6712.
12. van Dulmen, A. (2001). Pollination and phenology of flowers in the canopy of two contrasting rain forest types in Amazonia, Colombia. Plant Ecology *153*, 73-85.
13. L'Institut de recherche pour le développement en Guyane - http://www.cayenne.ird.fr/ - [Date accessed: 26th of January 2010]
14. Sasaki, D., Zappi, D., and Milliken, W. (2008). Vegetação do Parque Estudual Cristalino. Flora Cristalino.
15. Aluka - digital library of scholarly resources from and about Africa - http://www.aluka.org/ - [Date accessed: 26th of January 2010]
16. Wilkinson, H.P. (1989). Leaf Anatomy of the Menispermaceae Tribe Tiliacoreae Miers. Botanical Journal of the Linnean Society *99*, 125-174.
17. Schmelzer, G.H., and Gurib-Fakim, A. (2008). Plant Resources of Tropical Africa. Medicinal Plants. - Accessed from the World Wide Web on the 26th of January 2010: http://books.google.com/
18. Navone, S., Abraham, E., Bargiela, M., Dent, D.L., Espoz-Alsina, C., Maggi, A., Montana, E., Morrison, S., Pastor, G., Rosatto, H., et al. (2006). Global Deserts Outlook. Chapter 4: State and trends of the world's deserts. - Accessed from the World Wide Web on the 26th of January 2010: http://www.unep.org/Geo/gdoutlook/
19. Flowers in Israel - http://www.flowersinisrael.com/ - [Date accessed: 26th of January 2010]
20. Plant Biographies - http://www.plantlives.com/ - [Date accessed: 26th of January 2010]
21. Muthuramkumar, S., and Parthasarathy, N. (2001). Tree-liana relationships in a tropical evergreen forest at Varagalaiar, Anamalais, Western Ghats, India. Journal of Tropical Ecology *17*, 395-409.
22. Protabase - Webdatabase on useful plants of Tropical Africa - http://database.prota.org/ - [Date accessed: 26th of January 2010]
23. Mori, S.A., and Boom, B.M. (1987). Chapter II. The Forest. Mem. New York Botanical Gardens *44*, 9-29.
24. National Biodiversity Institute - Costa Rica - http://www.inbio.ac.cr/ - [Date accessed: 26th of January 2010]
25. National Plant Germplasm System - United States Department Of Agriculture - http://www.ars-grin.gov/npgs/ - [Date accessed: 26th of January 2010]
26. Carlquist, S. (2003). Wood and stem anatomy of woody Amaranthaceae s.s.: ecology, systematics and the problems of defining rays in dicotyledons. Botanical Journal of the Linnean Society *143*, 1-19.
27. Conservatoire et jardin botaniques - Ville de Genève - Databases - http://ville-ge.ch/musinfo/bd/cjb/ - [Date accessed: 26th of January 2010]
28. Ben Hassine, A., Ghanem, M.E., Bouzid, S., and Lutts, S. (2008). An inland and a coastal population of the Mediterranean xero-halophyte species *Atriplex halimus* L. differ in their ability to accumulate proline and glycinebetaine in response to salinity and water stress. Journal of Experimental Botany *59*, 1315-1326.
29. Carlquist, S. (1977). Ecological Factors in Wood Evolution - Floristic Approach. American Journal of Botany *64*, 887-896.
30. Growing Native Plants - Australian National Botanic Gardens - http://www.anbg.gov.au/gnp/ - [Date accessed: 26th of January 2010]
31. Spalding, M., Blasco, F., and Field, C. (1997). World Mangrove Atlas. Okinawa: The International Society for Mangrove Ecosystems.
32. Tomlinson, P.B. (1994). The Botany of Mangroves. Cambridge: Cambridge University Press.
33. Philipson, W.R., Ward, J.M., and Butterfield, B.G. (1971). Anomalous cambia. In The vascular cambium. Its development and activity., 1st Edition. (Chapman & Hall LTD).
34. Duke, N.C. (1991). A systematic revision of the mangrove genus *Avicennia* (Avicenniaceae) in Australasia. Aust. Syst. Bot. *4*, 299-324.
35. Gill, A.M. (1971). Endogenous control of growth-ring development in *Avicennia*. Forest Science *17*, 462-465.
36. Zamski, E. (1979). The mode of secondary growth and the three-dimensional structure of the phloem in *Avicennia*. Botanical Gazette *140*, 67-76.
37. Zamski, E. (1981). Does Successive Cambia Differentiation in *Avicennia* Depend on Leaf and Branch Initiation. Israel Journal of Botany *30*, 57-64.
38. Duke, N.C. (2006). Australia's mangroves. The authorative guide to Australia's mangrove plants. Brisbane: University of Queensland.
39. Sun, Q., and Suzuki, M. (2000). Wood anatomy of mangrove plants in Iriomote Island of Japan: a comparison with mangrove plants from lower lattitudes. Acta Phytotax. Geobot. *51*, 37-55.

40. Sun, Q., and Suzuki, M. (2001). Quantitative character variations of cambial derivatives in mangroves and their functional significance. Trees-Structure and Function *15*, 249-261.
41. Baker, R.T. (1915). The Australian "grey mangrove," (*Avicennia officinalis*, Linn.). J. Proc. roy. Soc. NS Wales *49*, 257-281.
42. Carlquist, S. (2002). Wood and bark anatomy of Salvadoraceae: ecology, relationships, histology of interxylary phloem. Journal of the Torrey Botanical Society *129*, 10-20.
43. Carlquist, S. (1999). Wood anatomy, stem anatomy, and cambial activity of *Barbeuia* (Caryophyllales). Iawa Journal *20*, 431-440.
44. The families of flowering plants: descriptions, illustrations, identification, and information retrieval - http://delta-intkey.com - [Date accessed: 26th of January 2010]
45. eFloras - http://efloras.org/ - [Date accessed: 26th of January 2010]
46. Kubitzki, K., Rohwer, J.G., and Bittrich, V. (1993). The Families and Genera of Vascular Plants. Volume VII: Flowering plants - Dicotyledons: Magnoliid, Hamamelid, and Caryophyllid families. - Accessed from the World Wide Web on the 26th of January 2010: http://books.google.com/
47. Bhambie, S. (1972). Correlation between form, structure and habit in some lianas. Proceedings of the Indian Academy of Sciences *75*, 246-256.
48. Carlquist, S. (2004). Lateral meristems, successive cambia and their products: a reinterpretation based on roots and stems of Nyctaginaceae. Botanical Journal of the Linnean Society *146*, 129-143.
49. Plants Database - Natural Resources Conservation Service - United States Department of Agriculture - http://www.plants.usda.gov/ - [Date accessed: 26th of January 2010]
50. Blue Planet Biomes - http://www.blueplanetbiomes.org/ - [Date accessed: 26th of January 2010]
51. Lüdtke, R., de Souza-Chies, T.T., and Miotto, S.T.S. (2008). Bredemeyera Willd. e Securidaca L. (Polygalaceae) na Região Sul do Brasil. Revista Brasileira De Biociênias *6*.
52. Hecklau, E.F., Mori, S.A., and Brown, J.L. (2005). Specific epithets of the flowering plants of central French Guiana. Brittonia *57*, 68-87.
53. Peel, M.C., Finlayson, B.L., and McMahon, T.A. (2007). Updated world map of the Koppen-Geiger climate classification. Hydrology and Earth System Sciences *11*, 1633-1644.
54. National Park Service - U.S. Department of the Interior - http://www.nps.gov/ - [Date accessed: 26th of January 2010]
55. Heddle, M.L., Wood, K.R., Asquith, A., and Gillespie, R.G. (2000). Conservation status and research on the Fabulous Green Sphinx of Kaua'i, *Tinostoma smaragditis* (Lepidoptera: Sphingidae), including checklists on the vascular plants of the diverse mesic forests of Kaua'i, Hawai'i. Pacific Conservation *54*, 1-9.
56. Federal Register Environmental Documents - U.S. Environmental Protection Agency - http://www.epa.gov/fedrgstr/ - [Date accessed: 26th of January 2010]
57. SysTax - a Database System for Systematics and Taxonomy - http://www.biologie.uni-ulm.de/systax/ - [Date accessed: 26th of January 2010]
58. Mennega, A.M.W. (1997). Wood anatomy of the Hippocrateoideae (Celastraceae). Iawa Journal *18*, 331-368.
59. Botanicus - http://www.botanicus.org/ - [Date accessed: 26th of January 2010]
60. Neotropical Herbarium Specimens - Fieldmuseum - http://fm1.fieldmuseum.org/vrrc/ - [Date accessed: 26th of January 2010]
61. Atrium - Biodiversity Information System - http://atrium.andesamazon.org/ - [Date accessed: 26th of January 2010]
62. Pagina dos colecionadores de frutas - http://frutasraras.sites.uol.com.br/ - [Date accessed: 26th of January 2010]
63. Gonçalves-Esteves, V., and Sant'Anna Melhem, T. (2004). Palynotaxonomy of the Brazilian species of *Cheiloclinium* Miers (Hippocrateaceae Juss.). Acta Botanica Brasilica *18*.
64. Senock, R.S., and Taylor, S.A. (2003). *Chenopodium oahense* (College of Agriculture, Forestry and Natural Resource Management, University of Hawaii). - Accessed from the World Wide Web on the 26th of January 2010: http://www.fs.fed.us/global/iitf/pdf/shrubs/Chenopodium%20oahuenseFinallEd2.pdf
65. Metabolomics - http://www.metabolomics.jp - [Date accessed: 26th of January 2010]
66. Rajput, K.S., and Rao, K.S. (2003). Cambial variant and xylem structure in the stem of *Cocculus hirsutus* (Menispermaceae). Iawa Journal *24*, 411-420.
67. Friis, I., and Vollesen, K. (2005). Flora of the Sudan-Uganda border area east of the Nile. - Accessed from the World Wide Web on the 26th of January 2010: http://books.google.com/
68. Biodiversity Informatics and co-Operation in Taxonomy for Interactive shared Knowledge base (BIOTIK) - http://www.biotik.org/ - [Date accessed: 26th of January 2010]
69. Iwu, M.M. (1993). Handbook of African medicinal plants. - Accessed from the World Wide Web on the 26th of January 2010: http://books.google.com/
70. De Feo, V., Piacente, S., Pizza, C., and Soria, R.U. (1998). Saponins from *Colignonia scandens* Benth. (Nyctaginaceae). Biochemical Systematics and Ecology *26*, 251-253.
71. Simon, G., Dewelle, J., Nacoulma, O., Guissou, P., Kiss, R., Daloze, D., and Braekman, J.C. (2003). Cytotoxic pentacyclic triterpenes from *Combretum nigricans*. Fitoterapia *74*, 339-344.
72. Ortiz-Gentry, R.d.C. (1999) Systematic revision of *Curarea* Barneby & Krukoff (Menispermaceae). Missouri, St Louis M. Sc.
73. Krishnamurthy, K.V., and Venugopal, N. (1984). Studies on successive cambia of a few plants. Indian J. Bot. *7*, 183-189.
74. Yang, M.G., and Mickelse.O (1968). Cycad Husk from Guam - Its Toxicity to Rats. Economic Botany *22*, 149-&.

75. ARKive - Images of Life on Earth - http://www.arkive.org/ - [Date accessed: 26th of January 2010]
76. Palm and Cycad Societies of Australia (PACSOA) - http://www.pacsoa.org.au/ - [Date accessed: 26th of January 2010]
77. The Gymnosperm Database - http://www.conifers.org/ - [Date accessed: 26th of January 2010]
78. Terrazas, T. (1991). Origin and Activity of Successive Cambia in *Cycas* (Cycadales). American Journal of Botany *78*, 1335-1344.
79. Flowers of India - http://www.flowersofindia.net/ - [Date accessed: 26th of January 2010]
80. Whitelock, L.M. (2002). The Cycads. - Accessed from the World Wide Web on the 26th of January 2010: http://books.google.com/
81. New South Wales Flora Online - Botanic Gardens Trust - Sydney Australia - http://plantnet.rbgsyd.nsw.gov.au/ - [Date accessed: 26th of January 2010]
82. Kodandapani, N., Cochrane, M.A., and Sukumar, R. (2008). A comparative analysis of spatial, temporal, and ecological characteristics of forest fires in seasonally dry tropical ecosystems in the Western Ghats, India. Forest Ecology and Management *256*, 607-617.
83. Nair, M.N.B., and Ram, H.Y.M. (1990). Structure of Wood and Cambial Variant in the Stem of *Dalbergia paniculata* Roxb. Iawa Bulletin *11*, 379-391.
84. Okafor, J. (1999). The use of farmer knowledge in non-wood forest product research. In: Current research issues and prospects for conservation and development. - Accessed from the World Wide Web on the 26th of January 2010: http://www.fao.org/documents/
85. Standley, P.C., Williams, L.O., and Gibson, D.N. (1970). Flora of Guatemala. Fieldiana: Botany *24*.
86. The International Plant Names Index - http://www.ipni.org/ - [Date accessed: 26th of January 2010]
87. Carlquist, S. (2000). Wood and stem anatomy of phytolaccoid and rivinoid Phytolaccaceae (Caryophyllales): ecology, systematics, nature of successive cambia. Aliso *19*, 13-29.
88. dos Anjos, L., Volpato, G.H., Lopes, E.V., Serafini, P.P., Poletto, F., and Aleixo, A. (2007). The importance of riparian forest for the maintenance of bird species richness in an Atlantic Forest remnant, southern Brazil. Revista Brasileira De Zoologia *24*, 1078-1086.
89. Markesteijn, L., Poorter, L., and Bongers, F. (2007). Light-dependent leaf trait variation in 43 tropical dry forest tree species. American Journal of Botany *94*, 515-525.
90. Petit Lobão, E.V., and Valeri, S.V. (2007). Agroecossistema cacaueiro da bahia: cacaucabruca e fragmentos florestais na conservação de espécies arbóreas. Jaboticabal.
91. Augustine, A.C., and DSouza, L. (1997). Somatic embryogenesis in *Gnetum ula* Brongn (*Gnetum edule*) (Willd) Blume. Plant Cell Reports *16*, 354-357.
92. Wikipedia - The Free Encyclopedia - http://wikipedia.org/ - [Date accessed: 26th of January 2010]
93. Endlicher, S.L.F. (1847). Synopsis Coniferarum. - Accessed from the World Wide Web on the 26th of January 2010: http://books.google.com/
94. Wild World - http://www.worldwildlife.org/wildworld/ - [Date accessed: 26th of January 2010]
95. Reddy, S.M. (2003). University Botany 2. Gymnosperms, Plant Anatomy, Genetics, Ecology. - Accessed from the World Wide Web on the 26th of January 2010: http://books.google.be/
96. Merrill, E.D. (1918). New or noteworthy Philippine plants. The Philippine Journal of Science *13(1)*, January.
97. Al-Khalifah, N.S., and Shanavaskhan, A.E. (2007). On the distrubution, status and phenology of Ghada (*Haloxylon persicum* Bunge) in the Arabian Peninsula. Tropical Ecology *48*, 51-60.
98. Warren, A. (1981). Arid-Land Ecosystems - Structure, Functioning and Management, Vol 1 - Goodall,Dw, Perry,Ra. Geographical Journal *147*, 215-217.
99. Flora de la Region del Parque National Amboro - http://www.nybg.org/botany/nee/ - [Date accessed: 26th of January 2010]
100. ParksWatch - http://www.parkswatch.org/ - [Date accessed: 26th of January 2010]
101. DeWalt, S.J., Ickes, K., Nilus, R., Harms, K.E., and Burslem, D.F.R.P. (2006). Liana habitat associations and community structure in a Bornean lowland tropical forest. Plant Ecology *186*, 203-216.
102. Mcdonald, J.A. (1992). Evolutionary Implications of Typical and Anomalous Secondary Growth in Arborescent *Ipomoea* (Convolvulaceae). Bulletin of the Torrey Botanical Club *119*, 262-267.
103. Center for Sonoran Desert Studies - Arizona-Sonora Desert Museum - http://www.desertmuseum.org/center/ - [Date accessed: 26th of January 2010]
104. Llamas, K. (2003). Tropical Flowering Plants: A guide to identification and cultivation. - Accessed from the World Wide Web on the 26th of January 2010: http://ebook30.com/
105. Floridata - http://www.floridata.com - [Date accessed: 26th of January 2010]
106. Pérez-García, E.A., Meave, J., and Gallardo, C. (1988). Vegetatión y Flora de la regíon de Nizanda, istmo de Tehuantepec, Oaxaca, México. Acta Botanica Mexicana *56*.
107. NewCROP - Center for New Crops & Plant Products - Purdue University - http://www.hort.purdue.edu/newcrop/ - [Date accessed: 26th of January 2010]

108. Parra-Tabla, V., and Bullock, S.H. (2005). Ecological and selective effects of stigma-anther separation in the self-incompatible tropical tree *Ipomoea wolcottiana* (Convolvulaceae). Plant Systematics and Evolution *252*, 85-95.
109. Forest Department Sarawak - http://www.forestry.sarawak.gov.my/ - [Date accessed: 26th of January 2010]
110. Leon-Gomez, C., and Monroy-Ata, A. (2005). Seasonality in cambial activity of four lianas from a Mexican lowland tropical rainforest. Iawa Journal *26*, 111-120.
111. Discover Life - http://www.discoverlife.org/ - [Date accessed: 26th of January 2010]
112. Carlquist, S. (2007). Successive cambia in Aizoaceae: products and process. Botanical Journal of the Linnean Society *153*, 141-155.
113. Croat, T.B. (1978). Flora of Barro Colorado Island. - Accessed from the World Wide Web on the 26th of January 2010: http://books.google.com/
114. Schmitz, E., Lötter, M., and McCleland, W. (2002). Trees and shrubs of Mpumalanga and Kruger National Park. - Accessed from the World Wide Web on the 26th of January 2010: http://books.google.com/
115. Palmer, T. (2004). Vegetation of Makana. Makana LEAP: Comprehensive Environmental AUdit - Accessed from the World Wide Web on the 26th of January 2010: https://www.ru.ac.za/documents/Environment/b%20Vegetation%20of%20Makana.pdf
116. Hawaiian Native Plant Propagation Database - College of Tropical Agriculture and Human Resources - University of Hawai at Manoa - http://pdcs.ctahr.hawaii.edu:591/hawnprop/ - [Date accessed: 26th of January 2010]
117. Gillespie, L.J., Hollowell, T., Funk, V.A., and Kelloff, C.L. (2003). Smithsonian Plant Collections, Guyana: 1989-1991, Lynn J. Gillespie. Contributions from the United States National Herbarium *44*, 1-104.
118. Kabir, M.E., and Webb, E.L. (2006). Saving a forest: the composition and structure of a deciduous forest under community management in northeast Thailand. Nat. Hist. Siam Soc. *54*, 239-260.
119. MacBride, J.F. (1961). Flora of Peru. Botanical Series XIII (1). - Accessed from the World Wide Web on the 26th of January 2010: http://www.archive.org/stream/floraofperueuphofimacb/floraofperueuphofimacb_djvu.txt
120. Léon, B., Pitman, N., and Roque, J. (2006). Introducción a las plantas endémicas del Perú. Rev. Peru. Biol. Numero especial *13*, 9-22.
121. Lombardi, J.A., and Temponi, L.G. (1999). A new species of *Peritassa* Miers (Hippocrateaceae) from Southern Brazil, and notes on two confused species. Novon *9*, 221-226.
122. de Araújo, D.S.D., and contributors, a. (2009). Área de proteçao ambiental de Massambaba, Rio de Janeiro: caracterização fitofisionômica e florística. Rodriguésia *60*, 67-96.
123. Smithsonian Tropical Research Institute Herbarium - http://biogeodb.stri.si.edu/herbarium/ - [Date accessed: 26th of January 2010]
124. Carlquist, S. (1998). Wood and stem anatomy of Petiveria and Rivina (Caryophyllales); systematic implications. Iawa Journal *19*, 383-391.
125. Garcia-Mateos, M.R., Sanchez, E.E., Espinosa-Robles, P., and Alvarez-Sanchez, M.E. (2007). Toxicity of Petiveria alliacea L. on Greenhouse Whitefly (Trialeurodes vaporariorum West.). Interciencia.
126. Gargiullo, M.B., Magnuson, B.L., and Kimball, L.D. (2008). A field guide to plants of Costa Rica. - Accessed from the World Wide Web on the 26th of January 2010: http://books.google.com/
127. Wheat, D. (1977). Successive Cambia in Stem of *Phytolacca dioica*. American Journal of Botany *64*, 1209-1217.
128. Esser, K.B., Semagn, K., and Wolde-Yohannes, L. (2003). Medicinal use and social status of the soap berry endod (Phytolacca dodecandra) in Ethiopia. Journal of Ethnopharmacology *85*, 269-277.
129. Friis, I., and Vollesen, K. (1998). Flora of the Sudan-Uganda Border Area East of the Nile: I. Catalogue of vascular plants, 1st part. - Accessed from the World Wide Web on the 26th of January 2010: http://books.google.com/
130. Wayne's Word - An On-line Textbook of Natural History - http://waynesword.palomar.edu/ - [Date accessed: 26th of January 2010]
131. Studholme, W.P., and Philipson, W.R. (1966). A comparison of the cambium in two woods with included phloem: *Heimerliodendron brunonianum* and *Avicennia resinifera*. New Zealand Journal of Botany *4*, 355-365.
132. Hawaiian Ethnobotany Online Database - http://www2.bishopmuseum.org/ethnobotanydb/index.asp - [Date accessed: 26th of January 2010]
133. Rodríguez, R.R. (2001). Variation in *Polygala guantanamana* (Polygalaceae), a cuban endemic species. Willdenowia *31*.
134. Google Directory / Flora and Fauna - http://directory.google.com/Top/Science/Biology/Flora_and_Fauna/ - [Date accessed: 26th of January 2010]
135. Carlquist, S. (2001). Wood and stem anatomy of Rhabdodendraceae is consistent with placement in Caryophyllales *sensu lato*. Iawa Journal *22*, 171-181.

136. Addo-Fordjour, P., Anning, A.K., Larbi, J.A., and Akyeampong, S. (2009). Liana species richness, abundance and relationship with trees in the Bobiri forest reserve, Ghana: Impact of management systems. Forest Ecology and Management *257*, 1822-1828.
137. TropHort - Life and Earth Sciences - http://trophort.com/ - [Date accessed: 26th of January 2010]
138. Plotkin, M.J., and Famolare, L. (1992). Sustainable harvest and marketing of rain forest products. - Accessed from the World Wide Web on the 26th of January 2010: http://books.google.com/
139. Smith, A.C. (1940). The American species of Hippocrateaceae. Brittonia *3*, 341-555.
140. Janick, J., and Paull, R.E. (2008). The Encyclopedia of Fruit & Nuts. - Accessed from the World Wide Web on the 26th of January 2010: http://books.google.com/
141. Levey, D.J., Silva, W.R., and Galetti, M. (2002). Seed dispersal and frugivory: ecology, evolution and conservation -Accessed from the World Wide Web on the 26th of January 2010: http://books.google.com/
142. Stanton, J.P., and Fell, D. (1940). The rainforests of Cape York Peninsula In Cooperative Research Centre for Tropical Rainforest Ecology and Management. p. 177.
143. Martins, V.L.C. (2005). Tipos das plantas vasculares do herbario do museu nacional (R) -XXIV: Gentianaceae, Haloragaceae, Hippocrateaceae, Humiriaceae, Icacinaceae, Verbenaceae, Violaceae e Vitaceae. Boletim do museu nacional. Botânica *126*.
144. Flora Zambesiaca - Kew Gardens - http://apps.kew.org/efloras/ - [Date accessed: 26th of January 2010]
145. Bongers, F., Charles-Dominique, P., Forget, P.-M., and Théry, M. (2001). Nouragues. Dynamics and plant-animal interactions in a neotropical rainforest. - Accessed from the World Wide Web on the 26th of January 2010: http://books.google.be
146. Hanelt, P., Büttner, R., and Mansfeld, R. (2001). Mansfeld's Encyclopedia of Agricultural and Horticultural Crops (except ornamentals). - Accessed from the World Wide Web on the 26th of January 2010: http://books.google.com/
147. Virtual Herbarium Berolinense - Freie Universität Berlin - http://ww2.bgbm.org/herbarium/ - [Date accessed: 26th of January 2010]
148. Poorter, L., Bongers, F., Koeamé, F.N., and Hawthorne, W.D. (2004). Biodiversity of West African Forests. An Ecological Atlas of Woody Plant Species -Accessed from the World Wide Web on the 26th of January 2010: http://books.google.com/
149. Missouri Botanical Garden - http://www.mobot.org - [Date accessed: 26th of January 2010]
150. Oliveira-Filho, A.T., and Ratter, J.A. (1994). Database: woody flora of 106 forest areas of eastern tropical South America. Occasional Monographs from the Royal Botanic Garden Edinburgh - Accessed from the World Wide Web on the 26th of January 2010: http://www.icb.ufmg.br/treeatlan/Downloads/t02.pdf
151. Khare, C.P. (2004). Indian Herbal Remedies. Rational Western Therapy, Ayurvedic and Other Traditional Usage, Botany. - Accessed from the World Wide Web on the 26th of January 2010: http://books.google.com/
152. AgroForestryTree Database - http://www.worldagroforestrycentre.org/SEA/Products/AFDbases/AF/ - [Date accessed: 26th of January 2010]
153. NatureServe Explorer - An Online Encyclopedia of Life - http://www.natureserve.org/explorer/ - [Date accessed: 26th of January 2010]
154. Plants For A Future - Edible, medicinal and useful plants for a healthier world - http://www.pfaf.org/ - [Date accessed: 26th of January 2010]
155. Nabe-Nielsen, J. (2001). Diversity and distribution of lianas in a neotropical rain forest, Yasuni National Park, Ecuador. Journal of Tropical Ecology *17*, 1-19.
156. Burnham, R.J. (2004). Alpha and beta diversity of Lianas in Yasuní, Ecuador. Forest Ecology and Management *190*, 43-55.
157. Graham, A., and Dilcher, D.L. (1998). Studies in neotropical paleobotany. XII. A palynoflora from the Pliocene Rio Banano Formation of Costa Rica and the Neogene vegetation of Mesoamerica. American Journal of Botany *85*, 1426-1438.
158. Fischer, A.F. (1921). Minor products from Philippine forests. Bulletin Nr 22 *III* - Accessed from the World Wide Web on the 26th of January 2010: http://www.archive.org/stream/minorproductsofp03brow/minorproductsofp03brow_djvu.txt
159. Acevedo-Rodríguez, P., and Axelrod, F.S. (1999). Annotated Checklist for the Tracheophytes of Río Abajo Forest Reserve, Puerto Rico. Carribean Journal of Science *35*, 265-285.
160. Oliveira-Filho, A.T., Jarenkow, L.A., and Rodal, M.J.N. (2006). Floristic relationships of seasonally dry forests of Eastern South America based on tree species distribution patterns. In: Neotropical Savannas and Seasonally Dry Forests: Plant Diversity, Biogeography, and Conservation. - Accessed from the World Wide Web on the 26th of January 2010: http://books.google.be/
161. Crabb, G. (1823). Universal technological dictionary on familiar explanation of the terms used in all arts and sciences. - Accessed from the World Wide Web on the 26th of January 2010: http://books.google.be

162. da Cunha, C.N., Junk, W.J., and Leitao-Filho, H.F. (2007). Woody vegetation in the Pantanal of Mato Grosso, Brazil: a preliminary typology. Amazoniana-Limnologia Et Oecologia Regionalis Systemae Fluminis Amazonas *19*, 159-184.
163. Fire Effects Information System - USDA Forest Service - http://www.fs.fed.us/database/feis/ - [Date accessed: 26th of January 2010]
164. Bailey, D.C. (1980). Anomalous Growth and Vegetative Anatomy of *Simmondsia chinensis*. American Journal of Botany *67*, 147-161.
165. The International Jojoba Export Council - http://www.ijec.net/ - [Date accessed: 26th of January 2010]
166. Utteridge, T., Nagamasu, H., Teo, S.P., White, L.C., and Gasson, P. (2005). *Sleumeria* (Icacinaceae): A new genus from northern Borneo. Systematic Botany *30*, 635-643.
167. Nair, M.N.B. (1993). Structure of Stem and Cambial Variant in *Spatholobus roxburghii* (Leguminosae). Iawa Journal *14*, 191-204.
168. Horak, K.E. (1981). Anomalous Secondary Thickening in *Stegnosperma* (Phytolaccaceae). Bulletin of the Torrey Botanical Club *108*, 189-197.
169. AllExperts - http://www.allexperts.com/ - [Date accessed: 26th of January 2010]
170. Sagebud - The Social Network For Gardeners - http://sagebud.com/ - [Date accessed: 26th of January 2010]
171. Carlquist, S. (1999). Wood and stem anatomy of *Stegnosperma* (Caryophyllales); phylogenetic relationships; nature of lateral meristems and successive cambial activity. Iawa Journal *20*, 149-163.
172. Ives, R.L. (1964). Vegetation and Flora of the Sonoran Desert - Shreve,F, Wiggins,Il. Journal of Geography *63*, 288-290.
173. Shreve, F., and Wiggins, I.L. (1964). Vegetation and Flora of the Sonoran Desert. Journal of Geography *63*, 288-290.
174. Pacific Island Ecosystems at Risk (PIER) - Institute of Pacific Islands Forestry - http://www.hear.org/pier/ - [Date accessed: 26th of January 2010]
175. Dobbins, D.R., and Fisher, J.B. (1986). Wound Responses in Girdled Stems of Lianas. Botanical Gazette *147*, 278-289.
176. BugwoodWiki - http://wiki.bugwood.org/ - [Date accessed: 26th of January 2010]
177. Tripathi, K.P., and Singh, B. (2009). Species diversity and vegetation structure across various strata in natural and plantation forests in Katerniaghat Wildlife Sanctuary, North India. Tropical Ecology *50*, 191-200.
178. Behrens, J.W., and Uhlworm, O. (2010). Botanisches Centralblatt; Referirendes Organ. - Accessed from the World Wide Web on the 26th of January 2010: http://books.google.be
179. Flora of Zimbabwe - http://www.zimbabweflora.co.zw/ - [Date accessed: 26th of January 2010]
180. ZipcodeZoo - http://zipcodezoo.com/ - [Date accessed: 26th of January 2010]
181. University of Florida Herbarium Collections Catalog - http://www.flmnh.ufl.edu/herbarium/cat/ - [Date accessed: 26th of January 2010]
182. Tannus, J.L.S., and Assis, M.A. (2005). Vascular species composition of dry and swamp grasslands in a savanna area, Itirapina - SP, Brazil. Revista Brasileira De Botânica *27*.
183. Global Biodiversity Information Facility - http://www.gbif.net/ - [Date accessed: 26th of January 2010]
184. Global Invasive Species Database - http://www.invasivespecies.net/database/ - [Date accessed: 26th of January 2010]
